# Single-cell transcriptome-guided biomimetic magnetothermal hydrogel microspheres for multimodal eradication of residual glioblastoma

**DOI:** 10.1016/j.bioactmat.2026.07.043

**Published:** 2026-07-28

**Authors:** Nan Wu, Xi Luan, Bihe Yuan, Mingyuan Xia, Jianliang Shen, Jing Lin, Lixia Xu, Hai Lin, Lexiang Zhang, Fangfu Ye, Hua Yan

**Affiliations:** a Department of Neurosurgery, Huanhu Hospital Affiliated to Tianjin Medical University, Tianjin Medical University, Tianjin, 300070, PR China; b Zhejiang Key Laboratory of Soft Matter Biomedical Materials, Wenzhou Institute, University of Chinese Academy of Sciences, Wenzhou, Zhejiang, 325000, PR China; c School of Physical Sciences, University of Science and Technology of China, Hefei, Anhui, 230026, PR China; d School of Materials Science and Engineering, Central South University, Changsha, Hunan, 410083, PR China; e Department of Obstetrics and Gynecology, The Second Affiliated Hospital and Yuying Children's Hospital of Wenzhou Medical University, Wenzhou, Zhejiang, 325027, PR China; f Tianjin Key Laboratory of Cerebral Blood Flow Reconstruction and Head and Neck Tumor New Technology Translation, Tianjin Neurosurgical Institute, Tianjin Huanhu Hospital, Tianjin, 300350, PR China; g Joint Centre of Translational Medicine, The First Affiliated Hospital of Wenzhou Medical University, Wenzhou, Zhejiang, 325035, PR China; h Beijing National Laboratory for Condensed Matter Physics, Institute of Physics, Chinese Academy of Sciences, Beijing, 100190, PR China; i Tianjin Fourth Central Hospital, The Affiliated Hospital of Tianjin Medical University, Tianjin, 300140, PR China

**Keywords:** Magnetothermal hydrogels, Droplet microfluidics, Single-cell RNA sequencing, Transcriptomic bioinformatics, Tumor microenvironments

## Abstract

Incomplete resection and rapid postoperative recurrence remain major challenges in glioblastoma (GBM) treatment. Inspired by the self-healing microduct architecture of pine resin, we developed an injectable magnetothermal-responsive hierarchical hydrogel microsphere platform, HGM + Multi(+), for spatiotemporally programmed magnetothermal ablation, anti-angiogenic blockade, and targeted chemotherapy. The system integrates T7-modified, pH-responsive temozolomide nanocarriers in GelMA microspheres with bevacizumab and Fe_3_O_4_ nanoparticles in a HAMA matrix, enabling rapid magnetothermal heating and sequential dual-drug release. Cellular and release studies demonstrated receptor-mediated uptake and tumor-microenvironment-responsive drug release. In orthotopic GBM resection models, a single intracavitary administration under alternating magnetic field markedly suppressed recurrence, reducing tumor volume to approximately 5% of controls and doubling median survival. In the immunocompetent postoperative GL261/C57BL/6J model, longitudinal IVIS, H&E-based tumor area quantification, and Kaplan–Meier analysis further confirmed reduced recurrent tumor burden and prolonged survival. Single-cell transcriptomic profiling of 47,781 cells revealed extensive tumor microenvironment remodeling, including macrophage polarization toward M1-like states, alleviated T cell exhaustion, enhanced cytotoxic programs, and an approximately 37% reduction in cancer stemness. These findings were further supported by ex vivo tumor-sphere assays and reduced Sox9/Nestin expression in recurrent tumors. Intercellular communication analysis indicated strengthened antigen-presentation signaling and intensified interactions between T cells and myeloid cells. Together, the integrated therapeutic, histological, and single-cell transcriptomic data support HGM + Multi(+) as a versatile postoperative strategy for eliminating residual GBM and advancing intelligent biomaterials for oncology.

## Highlights

• Spatiotemporal magnetothermal multimodal platform prevents recurrence in orthotopic GBM.
• Omics analysis deciphers the gene-level logic of immune microenvironment remodeling.
• ScRNA-seq reveals gene mechanisms of cold-to-hot tumor remodeling.

## Graphical abstract

**Figure:**
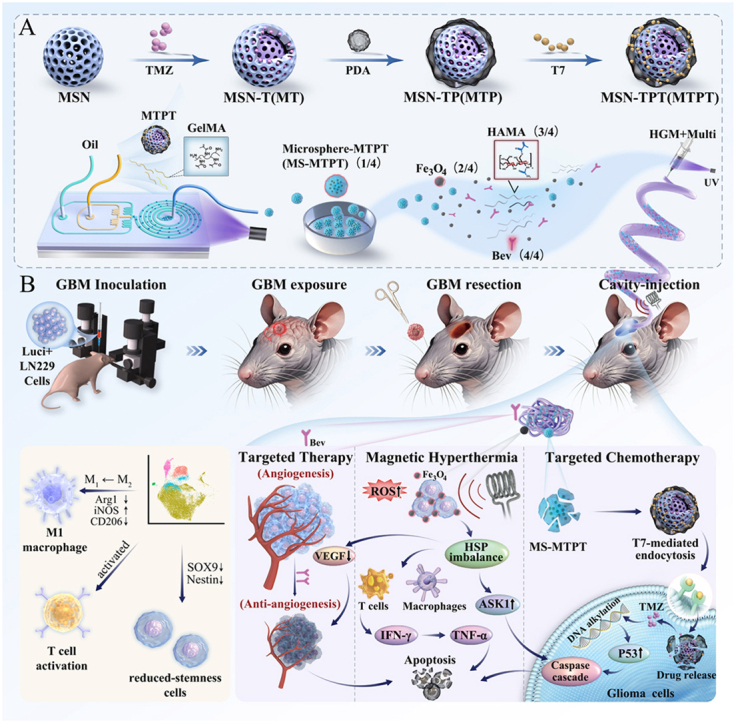

## 1 Introduction

Glioblastoma (GBM), the predominant and most aggressive malignant brain tumor, is primarily managed by maximal surgical resection followed by adjuvant radiotherapy and temozolomide (TMZ) [1,2]. Growing clinical consensus identifies recurrence prevention as the foremost objective of subsequent combination therapies. Currently, only two adjunct treatments have gained U.S. Food and Drug Administration approval: bevacizumab (Bev) for recurrent disease and tumor-treating fields for both newly diagnosed and relapsed cases [3]. Despite extensive clinical investigations involving repurposed and novel compounds, cell- and mRNA-based vaccines, emerging neoantigen-derived personalized peptide vaccines, and drug-agnostic strategies such as low-intensity pulsed ultrasound combined with systemically administered micron-sized bubbles [4,5], as well as photothermal therapy for near-infrared-triggered localized hyperthermia and tumor ablation [6], progress toward effective treatment has remained elusive. This stagnation is largely attributed to formidable obstacles posed by the blood-brain barrier, an immunosuppressive tumor microenvironment (TME), and the profound heterogeneity characteristic of GBM [7,8].

Given the inevitability of surgical resection and the resulting postoperative cavity, injectable hydrogel-based delivery systems [9] provide a suitable means to orchestrate the spatiotemporal release of bioactive payloads, ranging from small-molecule drugs and growth factors to stem cells, extracellular vesicles, and cell therapies [10]. Among these approaches, magnetothermal hydrogels are particularly appealing for deep-tissue in situ applications owing to their strong tissue penetration, biomimetic architecture, excellent biocompatibility, prolonged in vivo stability, and robust lesion-site retention. Critically, their adaptable physicochemical properties enable not only direct tumor cell eradication but also reshaping of the surrounding TME through immune cell recruitment and activation, analogous to an efficient antigen-presenting cell-mediated process that primes T cells and coordinates their trafficking and cytotoxic function [11].

Recent studies have further illustrated the potential of programmable nanosystems for targeted GBM therapy. For example, self-propelled nanobots driven by endogenous chemical gradients or external physical stimuli can migrate along H_2_O_2_ gradients within the TME and penetrate deeply into tumor tissues for targeted chemotherapy delivery [12,13]. Incorporating chemical recognition strategies, such as modulation of endothelial transporters, the use of penetrating peptides and lactoferrin, and tumor-targeting ligands such as folic acid and L-arginine, has further improved the adaptability, programmability, and biosafety of hydrogel-based modalities [14,15]. In this context, magnetothermal hydrogel platforms integrated with clinically relevant therapies highlight the potential of rationally engineered multimodal systems to overcome current therapeutic limitations while advancing mechanistic understanding of GBM treatment.

Inspired by the self-healing microducts of pine resin, characterized by injury-triggered release, adaptive sealing, solidification, and ion-responsive heating, we developed an injectable magnetothermal-responsive hierarchical hydrogel microsphere system for postoperative GBM therapy (Fig. 1). Droplet microfluidics uses discrete picoliter-to nanoliter-scale droplets as microreactors, enabling controlled compartmentalization, rapid mixing, and on-demand assembly of functional materials [[16], [17], [18]]. This approach affords excellent reproducibility, tunable mechanical properties, and efficient high-density loading, while allowing co-encapsulation of chemically diverse components, including hydrophilic and hydrophobic cargos [19,20]. Precisely engineered microchannels further support single-step, high-throughput fabrication of complex microsystems, including multicore, Janus, and layered architectures [21,22]. Such hybrid microsystems have shown broad therapeutic utility. For instance, magnetic Janus microrobots can accelerate tendon–bone healing through controlled ion release [23,24], whereas hydrogel microspheres can serve as biocompatible carriers for local delivery at GBM resection margins to suppress postoperative recurrence of residual tumor cells [25,26]. Similarly, alginate hydrogel microspheres have been used to transport extracellular vesicles for myocardial infarction repair [27].

**Fig. 1:**
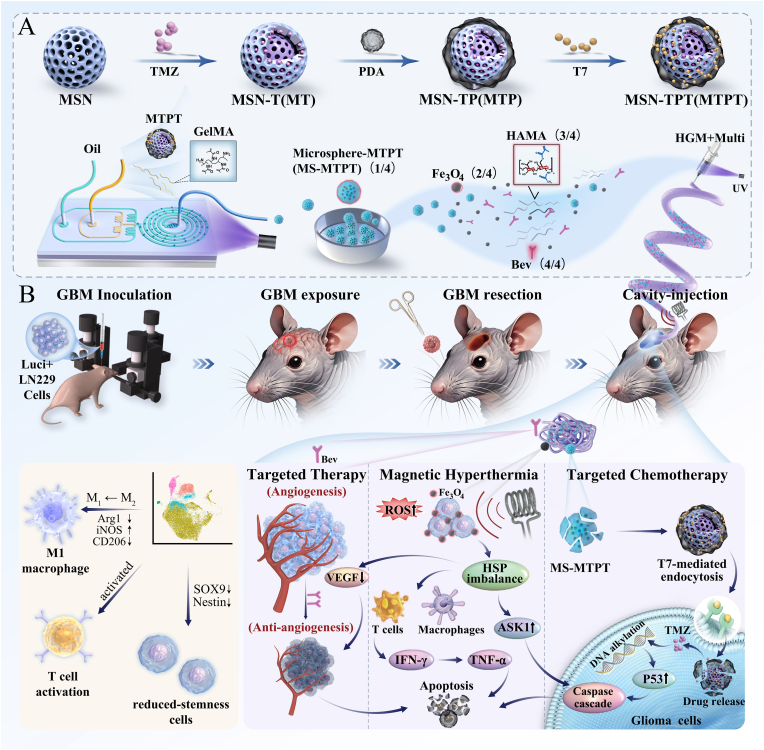
Fabrication, postoperative application, and therapeutic mechanism of HGM + Multi(+) for suppressing glioblastoma recurrence. (A) TMZ-loaded mesoporous silica nanoparticles were coated with PDA, functionalized with T7 peptide, encapsulated into GelMA microspheres via microfluidics, and further combined with Bev and Fe_3_O_4_ in a HAMA matrix to form the injectable hierarchical hydrogel system HGM + Multi(+). (B) Top: Luc^+^ LN229 cells were intracranially inoculated to establish orthotopic tumors, followed by surgical exposure and resection. The resulting cavity was then filled with HGM + Multi(+) for local postoperative treatment. Left: Single-cell transcriptomic analysis in the immunocompetent postoperative GL261/C57BL/6J model revealed treatment-associated remodeling of the tumor microenvironment. Right: HGM + Multi(+) suppresses postoperative recurrence through Bev-mediated anti-angiogenic blockade, Fe_3_O_4_-enabled magnetothermal stress, and T7-guided intracellular TMZ delivery, thereby promoting glioma cell apoptosis and recurrence control.

Meanwhile, single-cell omics and computational biology [28] have emerged as powerful tools for dissecting TME heterogeneity. In GBM, myeloid cells constitute nearly 50% of infiltrating populations and display distinct transcriptional programs that shift dynamically between immunosuppressive and pro-inflammatory states [29]. Single-cell analyses have further revealed that GBM recurrence frequently occurs at peritumoral high-blood-flow interfaces, where dense macrophage and T-cell infiltration defines critical therapeutic niches [30]. Although these technologies provide valuable benchmarks for evaluating synergistic therapeutic strategies, the immune circuitry governing postoperative GBM recurrence remains incompletely understood.

Herein, we developed a magnetothermal-responsive hierarchical hydrogel microsphere system, termed HGM + Multi(+), comprising a HAMA/GelMA/MSN-based local delivery platform co-loaded with Bev, TMZ, and Fe_3_O_4_ nanoparticles for postoperative GBM therapy. TMZ-loaded mesoporous silica nanoparticles were coated with polydopamine (PDA) and functionalized with the T7 peptide before encapsulation into GelMA microspheres, followed by integration with Fe_3_O_4_ nanoparticles and Bev within a HAMA matrix. In this design, superparamagnetic Fe_3_O_4_ serves as the magnetothermal core for alternating magnetic field-responsive heat generation and triggered drug release. PDA provides an interfacial layer for drug loading and biomolecular conjugation, while T7 peptide targets transferrin receptors overexpressed on GBM cells to enhance cellular uptake. Together with pH-responsive TMZ release and the local retention afforded by the hydrogel matrix, this platform enables coordinated postoperative chemo-, anti-angiogenic, and magnetothermal therapy. To assess both efficacy and mechanism, we combined orthotopic postoperative models with single-cell transcriptomic analysis under immunocompetent conditions. Additional in vivo validation in the same GL261/C57BL/6J postoperative model, including longitudinal tumor monitoring, histological evaluation, and survival assessment, was incorporated to support the biological relevance of the transcriptomic findings. Stemness-related changes were further examined using ex vivo tumor sphere formation and tissue-level marker analysis. Collectively, this study establishes a bioinspired multimodal hydrogel platform for local postoperative GBM treatment and provides insight into treatment-associated microenvironmental remodeling.

## 2 Methods

### 2.1 Materials

Chemicals and reagents, including Polydimethylsiloxane (PDMS), TMZ, Bev, and fluorescent dyes, were purchased from commercial vendors, including Sigma-Aldrich (St. Louis, MO, USA), Macklin (Shanghai, China), and Maladdin (Shanghai, China) and used as received. Gelatin methacryloyl (GelMA) and hyaluronic acid methacryloyl (HAMA) were obtained from Shuhe Biotechnology. Mesoporous silica nanoparticles (MSNs) were supplied by Zhichuan Technology, and the T7 peptide was synthesized by Hefei Peptide Library. Cell lines (LN229, U251, GL261, and human astrocytes) were obtained from accredited cell banks, authenticated by STR profiling within the last 3 years, and cultured in DMEM with 10% FBS.

### 2.2 Nanoparticle synthesis and characterization

TMZ-loaded MSNs (MT) were prepared by dispersing MSNs in a TMZ solution (10 mg mL^−1^) under stirring for 24 h. For PDA coating, MT particles were suspended in Tris-HCl buffer (pH 8.5) containing dopamine hydrochloride (2 mg mL^−1^) and reacted for 6 h under gentle stirring to allow oxidative self-polymerization of dopamine on the particle surface. Under these conditions, the particles retained well-preserved morphology without obvious excessive aggregation, as confirmed by SEM characterization. T7 peptide was subsequently conjugated onto the PDA-coated particles via Michael addition to yield MTPT. For fluorescence tracking, MTPT was labeled with 5-TAMRA-NHS ester. The physicochemical properties of the particles were characterized using TEM, SEM/EDS, FTIR, XPS, XRD, and TGA.

### 2.3 Microfluidic fabrication of MTPT-loaded microspheres

GelMA microspheres encapsulating MTPT (MS-MTPT) were fabricated using a flow-focusing PDMS microfluidic device. An aqueous phase (10% GelMA, 5% MTPT) and an oil phase (mineral oil, 1% Span 80) generated droplets that were photocrosslinked on-chip (365 nm, 10 mW/cm^2^) to ensure monodispersity. Microspheres were washed, freeze-dried, and sterilized.

### 2.4 Preparation of nanohybrid hydrogels (HGM + Multi)

HAMA (1% w/v) was dissolved in PBS and mixed with Bev (100 μg/mL), Fe_3_O_4_ nanoparticles (100 mM), and MS-MTPT (50 mg/mL). The precursor was partially photocrosslinked (365 nm, 20 s) to form an injectable filamentous hydrogel. Rheological properties (storage/loss moduli) were measured using a DHR-2 rheometer. Magnetothermal heating efficiency was assessed under an AMF (245 kHz, 6.2 A).

### *2.5* *In vitro* drug release and cellular assays

Release Kinetics: Cumulative release of TMZ and Bev from HGM + Multi was quantified by UV-Vis spectroscopy in PBS (pH 7.4, 6.0, and 5.0) with or without AMF activation. Hydrogel degradation was monitored gravimetrically. Cellular Uptake and Cytotoxicity: Glioma uptake of Tamra-labeled MTPT was visualized by confocal microscopy. Cytotoxicity was evaluated in a Transwell co-culture system using LN229 cells exposed to hydrogel formulations with or without AMF stimulation (15 min per session). Cell viability was assessed by CCK8 assay and Calcein AM/PI staining. Ex vivo tumorsphere formation assay: To evaluate treatment-associated changes in stem-like tumor growth, recurrent tumors from orthotopic postoperative GL261/C57BL/6J mice were harvested under sterile conditions and dissociated into single-cell suspensions. Primary tumor cells were cultured in serum-free neurosphere medium consisting of DMEM/F12 supplemented with B27, EGF, and bFGF under low-attachment conditions. Cells were maintained for sphere formation, and bright-field images were acquired at the indicated time points. Tumorsphere diameter was measured using ImageJ, and the average diameter from each sample was used for statistical analysis.

### 2.6 GBM mouse models and histology

All procedures using female BALB/c nude mice (4–6 weeks old, RRID: IMSR_CRL:022) and C57BL/6J mice (4–6 weeks old, RRID: IMSR_JAX:000664) obtained from the Zhejiang Provincial Center for Laboratory Animals were approved by the Ethical Committee of Wenzhou Institute, University of Chinese Academy of Sciences (WIUCAS24092301), and the study was conducted in compliance with the Public Health Service (PHS) Policy on Humane Care and Use of Laboratory Animals, ARRIVE Guidelines 2.0 (Percie du Sert et al., PLoS Biol, 2020), and the Laboratory Animal Care and Use Guidelines.

Mice were housed under specific pathogen-free (SPF) conditions with controlled temperature (22 ± 2 °C), humidity (50 ± 10%), and a 12-h light/dark cycle (3–5 mice per cage, ad libitum access to food and water) to ensure animal welfare. Prior to surgical procedures, mice were anesthetized with isoflurane (2% induction, 1% maintenance). For orthotopic implantation, a 1-mm burr hole was drilled 2 mm lateral and 1 mm posterior to the bregma, and cells were delivered stereotactically to a depth of 2 mm at 1 μL/min. The burr hole was sealed with bone wax and the scalp sutured. No preemptive analgesics were administered following specific ethical approval (Ref: WIUCAS24092301), because analgesic agents could potentially interfere with the neurological assessment endpoints of this study. Instead, mice were closely monitored for postoperative pain or distress (e.g., reduced activity, abnormal grooming, or impaired mobility).

For all intracranial tumor studies, mice were monitored regularly for humane endpoint criteria. Animals were euthanized when any of the following conditions were met: body weight loss exceeding 20% of the peak postoperative body weight; severe neurological symptoms indicative of progressive intracranial disease, including persistent ataxia, hemiplegia or limb paresis, or recurrent seizures; marked clinical deterioration such as hunching, impaired ambulation, or inability to access food or water; a moribund condition, defined as a near-terminal state characterized by minimal responsiveness, prolonged recumbency, or inability to maintain normal posture; or an intracranial bioluminescence signal exceeding a predefined disease-burden threshold of 1 × 10^8 p/sec/cm^2^/sr. Mice reaching endpoint criteria were euthanized by gradual fill CO_2_ inhalation (30% volume displacement per minute) followed by cervical dislocation, in accordance with the AVMA Guidelines for the Euthanasia of Animals.

Luc^+^ LN229 (xenograft) For the xenograft model, Luc^+^ LN229 cells (5 × 10^5^ in 5 μL PBS) were implanted as above. Tumor growth was monitored by bioluminescence imaging (BLI) using an IVIS Spectrum system after D-luciferin administration (75 mg/kg, i.p.). On day 10 post-implantation, tumors were microsurgically resected under an operating microscope. Immediately after resection, the surgical cavity was locally administered with the indicated formulation; specifically, mice in the PBS group received intracavitary PBS, whereas mice in all local treatment groups received intracavitary injection of the corresponding hydrogel formulation. In contrast, mice in the systemic injection group did not receive intracavitary administration, but instead received tail-vein injection of the indicated drugs. Mice were then randomized into nine groups using a random number table (randomization was performed by an investigator not involved in the subsequent experiments): 1) PBS; 2) HGM + TMZ + Bev + Fe_3_O_4_ (HGM + Multi); 3) HGM + TMZ + Bev + Fe_3_O_4_ with AMF (HGM + Multi(+); “(+)” denotes AMF ON); 4) systemic injection (TMZ 50 mg kg^−1^·day^−1^ + Bev 10 mg kg^−1^·day^−1^ via the tail vein for 3 consecutive days post-surgery); 5) HGM (blank); 6) HGM + Bev; 7) HGM + Fe_3_O_4_; 8) HGM + Fe_3_O_4_ with AMF (HGM + Fe_3_O_4_(+)); 9) HGM + TMZ. “(+)” denotes AMF exposure using a 100-mm-diameter, 6-turn coil at 245 kHz and 6.2 A for 15 min on postoperative days 1, 7, 14, and 21. The efficacy study follow-up ended on postoperative day 28.

Magnetothermal Mechanism Study: To assess the magnetothermal contribution to mono-therapy, a separate cohort underwent resection on day 10 and randomization into five groups (randomization was performed by an investigator not involved in the subsequent experiments): 1) PBS; 2) HGM + TMZ; 3) HGM + TMZ + Fe_3_O_4_(+) with AMF; 4) HGM + Bev; 5) HGM + Bev + Fe_3_O_4_(+) with AMF. Mice (n = 5 per group per time point) were perfused at pre-operation, day 1, week 1, week 2, week 3, and week 4.

Immune Profiling (GL261 Syngeneic Model): For the immunocompetent model, Luc^+^ GL261 cells were implanted as above. On day 10 post-implantation, tumors were resected and mice were randomized into two groups (n = 3 per group): 1) HGM + Multi(+) (HGM + TMZ + Bev + Fe_3_O_4_(+)) received AMF exposure using a 100-mm-diameter, 6-turn coil at 245 kHz and 6.2 A for 15 min on postoperative days 1, 7, 14, and 21; 2) systemic injection (TMZ 50 mg kg^−1^ day^−1^ + Bev 10 mg kg^−1^ day^−1^ via tail vein for 3 consecutive days post-surgery). All data collection and analysis were performed by investigators blinded to group assignments to minimize bias.

Efficacy Validation in the GL261 Syngeneic Model: To further evaluate therapeutic efficacy in an immunocompetent setting, an additional orthotopic Luc^+^ GL261 postoperative recurrence model was established in C57BL/6J mice. On day 10 post-implantation, tumors were resected and mice were randomized into three groups: 1) PBS; 2) Injection, defined as systemic administration of TMZ (50 mg kg^−1^ day^−1^) and Bev (10 mg kg^−1^ day^−1^) via the tail vein for 3 consecutive days after surgery; and 3) HGM + Multi(+) (HGM + TMZ + Bev + Fe_3_O_4_(+)) with AMF exposure using a 100-mm-diameter, 6-turn coil at 245 kHz and 6.2 A for 15 min on postoperative days 1, 7, 14, and 21. For longitudinal tumor burden monitoring, mice (n = 7 per group) underwent serial BLI using the IVIS Spectrum system after D-luciferin administration (75 mg/kg, i.p.), and signal intensity was quantified using a consistent cranial region of interest. For histological validation, brains (n = 5 mice per group) were collected on postoperative day 28 and subjected to H&E staining for tumor area quantification. Kaplan–Meier survival analysis was performed using the humane endpoint criteria defined above (n = 10 mice per group), and the date of euthanasia was recorded as the survival endpoint. All data collection and analysis were performed by investigators blinded to group assignments to minimize bias.

Histology: For the LN229 nine-arm study, mice (n = 5 per group) were perfused with saline followed by 4% paraformaldehyde (PFA) on day 28. For the magnetothermal add-on study, mice were perfused at the indicated time points. For the GL261 efficacy validation study, brains were harvested on postoperative day 28 for histological analysis. Brains and major organs were fixed in 4% PFA, paraffin-embedded, and sectioned. H&E staining was performed according to standard protocols. Briefly, paraffin sections were dewaxed to water, stained with Harris hematoxylin for nuclear visualization, differentiated in acid alcohol, blued in ammonia water, rinsed, and counterstained with eosin for cytoplasmic staining, followed by dehydration, clearing, and mounting with neutral resin. Proliferation and apoptosis were assessed by chromogenic immunohistochemistry (IHC) using Ki67 (1:500) and TUNEL (1:500) with DAB detection. Angiogenesis and proliferation in time-course samples were further evaluated by immunofluorescence staining using Ki67 (1:500) and CD31 (1:100), followed by FITC- and Texas Red-conjugated secondary antibodies. For stemness-marker analysis in recurrent orthotopic GL261 tumors, tumor sections were additionally subjected to immunofluorescence staining for Nestin and Sox9. After deparaffinization, rehydration, and antigen retrieval, sections were blocked with serum and incubated overnight at 4 °C with primary antibodies against Nestin and Sox9. After washing, sections were incubated with species-appropriate fluorescent secondary antibodies and counterstained with DAPI. Images were acquired using identical microscope settings on a Nikon A1 confocal microscope. For quantitative analysis, 3–5 randomly selected fields were analyzed for each tumor using ImageJ (version 1.54f), and the mean fluorescence intensity was normalized and averaged to generate one value per mouse. Histological and immunofluorescence images were analyzed using ImageJ (version 1.54f). Unless otherwise indicated, 3–5 regions of interest (ROIs) were analyzed per slide.

### 2.7 Local brain biocompatibility evaluation in normal mice

To evaluate the local brain tissue compatibility of the implanted hydrogel, a normal-brain implantation model was established in C57BL/6J mice by creating a small parenchymal cavity to simulate a postoperative tumor resection site. Briefly, mice were anesthetized and placed in a stereotaxic frame. After cranial drilling and dural exposure, a small cavity was generated in the brain parenchyma, followed by intracavitary administration of PBS or HGM + Multi hydrogel. In the HGM + Multi(+) group, mice received alternating magnetic field (AMF) stimulation using a 100-mm-diameter, 6-turn coil at 245 kHz and 6.2 A for 15 min on postoperative days 1, 7, 14, and 21.

At the indicated endpoint (6 weeks after implantation), mice were euthanized and perfused for brain tissue collection. Brains were fixed in 4% paraformaldehyde, paraffin-embedded, sectioned, and subjected to histological and immunofluorescence analyses. H&E staining was performed to assess general tissue architecture and possible local tissue damage surrounding the implantation cavity. To evaluate local neuroinflammatory and neuronal responses, sections were stained for GFAP, Iba-1, and NeuN. Fluorescence images were acquired under identical microscope settings, and quantitative analysis was performed using ImageJ. GFAP-positive area fraction, Iba-1-positive area fraction, and NeuN-positive neuronal density were quantified in peri-cavity regions of interest (ROIs). Data from 3 to 5 mice per group were included in the analysis.

### 2.8 Serum biochemistry, inflammatory cytokine assay, and ICP-MS analysis

Blood samples were collected from mice in the PBS, Injection, and HGM + Multi(+) groups at the indicated time points. Serum levels of alanine aminotransferase (ALT), aspartate aminotransferase (AST), uric acid (UA), and creatinine (Cr) were measured using a microplate reader (SpectraMax iD3, Molecular Devices, China) with commercially available assay kits (Nanjing Jiancheng Bioengineering Institute) according to the manufacturer's instructions. Serum TNF-α and IL-6 levels were quantified using ELISA kits following the manufacturer's protocols. For elemental analysis, brain, liver, and heart tissues were harvested at 6 weeks post-operation, digested, and analyzed for Fe content by inductively coupled plasma mass spectrometry (ICP-MS). Detailed procedures are provided in the Supplementary Information.

### 2.9 Single-cell RNA sequencing (scRNA-seq)

Tumors from GL261-bearing C57BL/6J mice (HGM + Multi(+) vs. Injection; n = 3 biologically independent mice/group) were harvested on day 28 for mechanistic single-cell transcriptomic profiling. Single-cell suspensions were processed using the 10x Genomics Chromium platform, and libraries were sequenced on an Illumina NovaSeq 6000. Sequencing data were processed using Cell Ranger and Seurat for quality control, clustering, integration, and cell-type annotation. Downstream analyses included trajectory inference (Monocle3), cell-cell communication (CellChat/CellPhoneDB), and differentiation state scoring (CytoTRACE). The scRNA-seq data were used to characterize treatment-associated cellular and transcriptional changes in the tumor microenvironment and were interpreted as mechanistic support in conjunction with histological and in vivo efficacy data. For each sample, cell-type annotations were assigned based on canonical marker genes and clustering results. The numbers of annotated immune-cell subsets were then extracted from the filtered scRNA-seq dataset and summarized for comparison across groups.

### 2.10 Statistical power and ethical considerations

All animal experiments were conducted in accordance with the ARRIVE guidelines and the ethical principles of the 3Rs (Replacement, Reduction, and Refinement). Sample sizes were selected according to the primary endpoint and the purpose of each experiment. For therapeutic efficacy studies, group sizes were determined to provide adequate power for detecting biologically meaningful differences in tumor burden and survival, and validation cohorts in this study generally ranged from n = 5 to 10 animals per group, depending on the experiment. For mechanistic analyses requiring intensive tissue-level characterization, including scRNA-seq and selected histological studies, n = 3 biologically independent mice per group were used. The scRNA-seq results were interpreted cautiously as mechanistic evidence and were not used as the sole basis for efficacy claims.

### 2.11 Statistical analysis

Data are presented as mean ± SD. Statistical comparisons were performed using GraphPad Prism 10.1.2. Two-tailed Student's t-tests were used for two-group comparisons. One-way ANOVA with Tukey's post-hoc test was used for multi-group comparisons at a single time point. Tumor growth curves were analyzed using two-way repeated-measures ANOVA with Bonferroni's correction. Survival distributions were compared using the Kaplan-Meier method and log-rank (Mantel-Cox) test. For survival analyses involving multiple groups, pairwise log-rank comparisons were performed as indicated, and exact P values are provided in the Supplementary Information where applicable. Quantification of fluorescence-based live/dead staining was performed using ImageJ from multiple representative microscopic fields per group. A P-value <0.05 was considered statistically significant. Statistical significance is indicated as ns, not significant; *P < 0.05; **P < 0.01; ***P < 0.001; ****P < 0.0001.

## 3 Results

### 3.1 Characterization of glioma targeted drug-loaded nanoparticles

The clinical efficacy of TMZ in GBM is constrained by poor penetration across the blood-brain barrier and insufficient uptake by tumor cells. Previous strategies employing hydrogel depots for peritumoral release did not achieve durable benefits, which was likely associated with limited intracellular delivery [31]. To address this, TMZ was encapsulated into MSN, a biocompatible and widely used nanocarrier system [32,33]. The nanoparticle surface was subsequently engineered to achieve glioma specific targeting and stimulus responsive drug release (Fig. 2A). Transmission electron microscopy (TEM) characterization revealed that pristine MSNs displayed a uniform spherical morphology with well-ordered internal mesopores and an average diameter of 226.8 nm. Energy-dispersive spectroscopy (EDS) verified silicon and oxygen as the predominant elemental constituents (Fig. 2B).

**Fig. 2:**
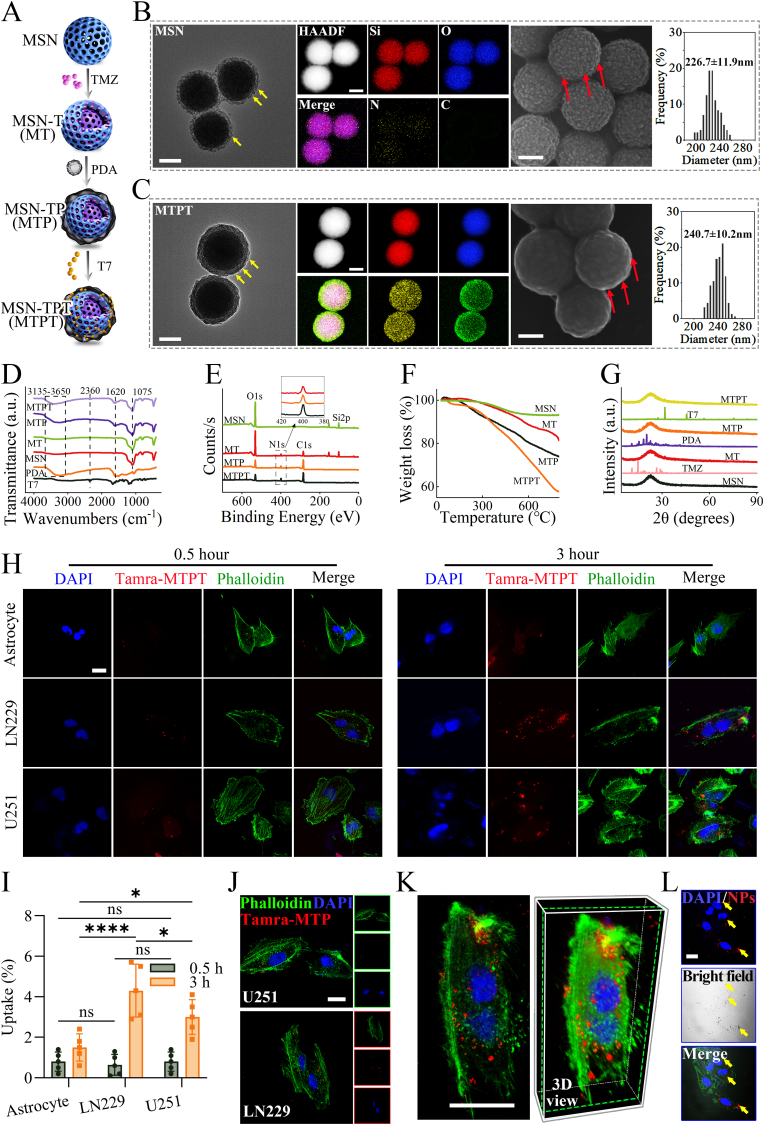
Characterization and cellular uptake of glioma-targeted, drug-loaded nanoparticles. (A) Schematic illustration of MSN-based nanoparticle fabrication, including TMZ loading, PDA coating, and T7 conjugation. (B, C) TEM, HAADF-EDS elemental mapping, SEM images, and particle size distributions of MSN (B) and MTPT (C). (D–G) FTIR spectra (D), XPS spectra (E), TGA curves (F), and XRD patterns (G) of MSN and functionalized nanoparticles. (H) Confocal images of human astrocytes, LN229, and U251 cells incubated with Tamra-labeled MTPT for 0.5 or 3 h. (I) Quantification of MTPT uptake in human astrocytes, LN229, and U251 cells after 0.5 or 3 h incubation. Data are mean ± SD. Statistical significance was analyzed by one-way ANOVA with Tukey's post hoc test. (J) Confocal images of LN229 and U251 cells incubated with Tamra-labeled MTP nanoparticles without T7 for 3 h. (K) Confocal and 3D reconstructed images of MTPT uptake in U251 cells. (L) Confocal, bright-field, and merged images confirming intracellular localization of MTPT nanoparticles in U251 cells. In (H) and (J–L), nuclei were stained with DAPI (blue), cytoskeleton with phalloidin (green), and nanoparticles are shown in red. Scale bars, 100 nm in (B, C) and 20 μm in (H, J–L).

To introduce functional conjugation sites and regulate pore accessibility, MSNs were coated with a polydopamine (PDA) layer to generate MSN-TMZ-PDA (MTP). Subsequent conjugation of the T7 targeting peptide yielded the final construct, MSN-TMZ-PDA-T7 (MTPT). Morphological characterization by TEM and scanning electron microscopy (SEM) revealed progressive surface coatings from MT to MTP, and finally to MTPT, consistent with sequential functionalization steps. Energy dispersive spectroscopy (EDS) mapping further identified the successful incorporation of carbon and nitrogen signals derived from TMZ, PDA, and T7 ligands. Particle sizing analysis showed a gradual increase in diameter from 231.9 nm for MT to 239.6 nm for MTP and 240.7 nm for MTPT. Despite the increase in diameter, all nanoparticle formulations maintained uniform morphology and narrow size distributions (Fig. 2B and C and S1).

Fourier-transform infrared spectroscopy (FTIR) confirmed the stepwise functionalization of the nanoparticle surface. MTP and MTPT displayed characteristic absorption bands at 3135-3650 cm^−1^ for N–H/O–H stretching, 1620 cm^−1^ for aromatic C–C stretching from the PDA coating, and 2360 cm^−1^ for C=O stretching attributable to the T7 peptide. In contrast, MT exhibited negligible spectral changes compared with pristine MSN, indicating only the physical encapsulation of TMZ (Fig. 2D). X-ray photoelectron spectroscopy (XPS) provided additional evidence of successful surface modification. N1s signals at 397–403 eV, which were absent in pristine MSN, emerged in MT, MTP, and MTPT samples and increased progressively with each modification step. The O1s binding energy shifted stepwise, while the disappearance of Si2p signals in MTP and MTPT indicated complete surface shielding by the organic layers. TMZ-derived N1s and C1s signals were clearly detected in MT, confirming efficient drug loading (Fig. 2E). Thermogravimetric analysis (TGA) demonstrated weight losses of 7.18% for pristine MSN, 18.71% for MT, 25.97% for MTP, and 42.08% for MTPT at 800 °C. Based on these thermal degradation profiles, the calculated loading contents were 11.53% for TMZ, 7.26% for PDA, and 16.11% for the T7 peptide (Fig. 2F). Wide-angle X-ray diffraction (XRD) analysis revealed that the crystalline TMZ peaks observed in the free drug disappeared after encapsulation, indicating the amorphization of the drug within the silica mesopores (Fig. 2G). Collectively, the FTIR, XPS, TGA, and XRD analyses corroborate efficient TMZ loading, uniform PDA coating, and effective T7 conjugation.

Given the overexpression of transferrin receptors (TfR) in glioma cells, we systematically assessed the targeting efficiency and cellular selectivity of the MTPT nanocomposite. Both MTPT and its non–T7-functionalized counterpart (MTP) were labeled with the fluorescent dye Tamra to visualize intracellular uptake dynamics. Confocal laser scanning microscopy (CLSM) revealed that MTPT readily penetrated LN229 and U251 glioma cells, whereas MTP exhibited negligible internalization (Fig. 2H and J). Both 3D confocal reconstruction and bright-field overlay imaging clearly confirmed that MTPT particles were internalized into glioma cells rather than remaining attached to the cell membrane surface (Fig. 2K and L). Time-resolved imaging further demonstrated a pronounced increase in intracellular MTPT fluorescence intensity during incubation. The fluorescence signal increased more than 6.7 folds between 0.5 h and 3 h (Fig. 2H and I). In contrast, human astrocytes exhibited minimal fluorescence signals throughout the observation period, indicating negligible off-target uptake. Collectively, these data support T7-dependent, preferential uptake of MTPT in GBM cells.

### 3.2 Pine resin inspired hierarchical nanohybrid hydrogel assembly and characterization

To achieve spatiotemporally controlled delivery, a hierarchical hydrogel platform was constructed through a two-step microfluidic assembly process. First, polydimethylsiloxane (PDMS) microfluidic chips were designed to generate monodisperse GelMA microspheres encapsulating MTPT nanoparticles (MS-MTPT). The device utilized a flow-focusing geometry where an aqueous GelMA phase containing MTPT interacted with a continuous mineral oil phase to generate water-in-oil droplets at frequencies exceeding 1000 Hz. A downstream annular parking channel facilitated on-chip UV photopolymerization, effectively preventing aggregation and overcoming the limitations associated with conventional bulk emulsification methods (Fig. 3A–C). Control of droplet size was achieved by tuning the oil-to-water flow rate ratio (Vo/Vw) and channel height (H). Increasing Vo/Vw from 1 to 6 expanded the microsphere diameter from 98.5 to 155.6 μm. We selected microspheres of approximately 145 μm diameter for subsequent integration. High-speed imaging confirmed the droplet formation mechanism, showing shear-induced breakup of the nanoparticle-laden GelMA stream by the oil phase (Fig. 3D). CLSM confirmed the uniform distribution of Tamra-labeled MTPT within the GelMA matrix, while SEM images showed that the microspheres possessed intact spherical morphology with interconnected internal porous structures that support sustained drug retention (Fig. 3E and F).

**Fig. 3:**
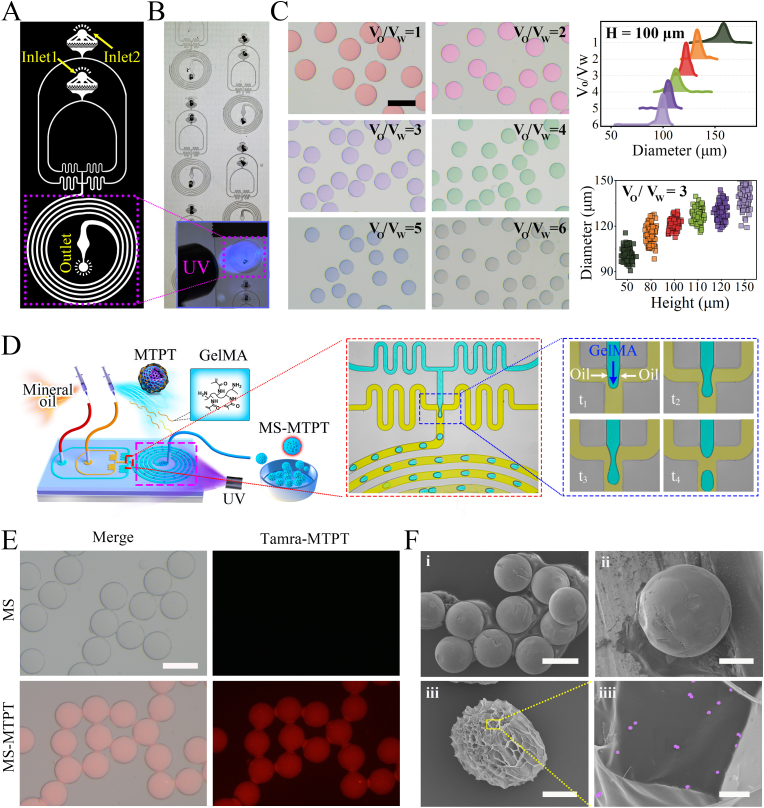
Tunable microfluidic generation of GelMA microspheres and MTPT loading. (A) Schematic design of the microfluidic chip. (B) Photograph of the fabricated chip, with an inset showing the UV-curing region within the microfluidic setup. (C) Bright-field images of GelMA microspheres prepared at different oil-to-water flow ratios, their corresponding size distributions (represented by ridge plots), and the dependence of microsphere size on channel height (H) under a fixed flow ratio (represented by scatter plots). (D) Mechanism of droplet formation. Inset: Shearing of the aqueous phase by the oil phase. (E) Bright-field and fluorescence images of blank microspheres and MTPT-loaded microspheres. (F) SEM images of the microspheres. Scale bars: 200 μm in (C) and (E); 50 μm in (F-i); 20 μm in (F-ii and iii); 2 μm in (F-iiii).

Microspheres with an average diameter of approximately 145 μm were selected to balance injectability, fabrication stability, and structural uniformity within the microfluidic system. This size allows smooth administration through standard 26G clinical needles while minimizing risks of clogging or particle deformation during hydrogel assisted injection. In addition, microspheres within the range of 120–160 μm are commonly used in microfluidic biomaterial fabrication because they exhibit stable droplet generation and reproducible structural characteristics. Within this size range, variations in specific surface area are relatively limited and are not expected to substantially influence the overall therapeutic performance or release profile of the platform.

Following microsphere fabrication, the cured MS-MTPT microspheres were incorporated into a HAMA matrix together with Bev and Fe_3_O_4_ nanoparticles to form the nanohybrid hydrogel (HGM + Multi). TEM showed that the Fe_3_O_4_ nanoparticles displayed a narrow particle size distribution of approximately 13 nm and exhibited superparamagnetic behavior (Fig. S2). The resulting composite maintained excellent injectability and could be extruded as continuous filaments capable of filling irregular surgical cavities (Fig. 4A). This hierarchical structure mimics the microduct structure of pine resin. The HAMA network provides structural filling of the resection cavity, while the embedded microspheres function as therapeutic reservoirs. SEM imaging revealed a distinct microstructural organization where the pristine HAMA network exhibited large lyophilization pores, whereas HGM + Multi displayed smaller and more uniformly distributed pores (Fig. 4B). The reduced pore size is advantageous for suppressing burst release of Bev. To optimize the Fe_3_O_4_ content, formulations containing 30, 70, 100, and 150 mM Fe_3_O_4_ were further compared in terms of magnetothermal performance and extrusion behavior. Fe_3_O_4_ at 100 mM was selected as the working concentration because it enabled the hydrogel to reach the mild-hyperthermia window (≈42–45 °C) within 3 min under AMF exposure while maintaining acceptable extrusion behavior. By contrast, lower concentrations (30 and 70 mM) failed to reach this temperature range, whereas the 150 mM formulation, despite stronger heating, showed less favorable extrusion morphology (Fig. S3A and B).

**Fig. 4:**
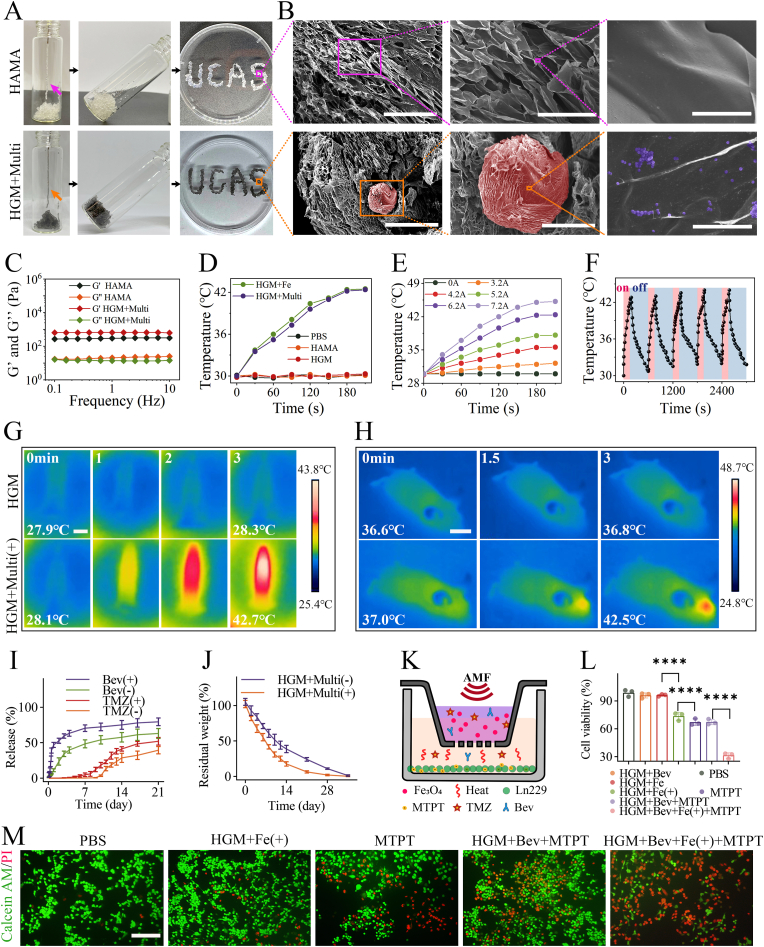
Properties and antitumor performance of nanohybrid hydrogels. (A) Injectability of HAMA and HGM + Multi hydrogels. (B) SEM images of HAMA and HGM + Multi matrices. (C) Frequency-sweep rheology of HGM + Multi and HAMA. (D) Magnetothermal heating profiles under AMF conditions with a current of 6.2 A and a frequency of 245 kHz using a coil with a diameter of 100 mm and six turns. (E) Heating curves of HGM + Multi at varying AMF power. (F) Temperature cycling under AMF pulses with 200 s activation and 400 s interval using the same parameters as in panel D. (G) Infrared thermal imaging of HAMA and HGM + Multi(+) under AMF (AMF parameters as in panel D). (H) Intracranial thermographic imaging of HGM + Multi(+) and HAMA after implantation under AMF stimulation. (I) Dual-drug release kinetics with AMF cycling. (J) Degradation profiles under AMF pulses. (K) Schematic illustration of hydrogel mediated tumor cell interactions. (L) Viability of LN229 cells treated with the indicated components with or without AMF (CCK-8 assay, 24 h). (M) Live/dead staining of LN229 cells treated with indicated components with or without AMF for 24 h. All data are mean ± SD (n = 3). Scale bars: 300 μm in (B left column), 100 μm in (B middle column), and 5 μm in (B right column), 5 mm in (G), 10 mm in (H), and 100 μm in (M).

Rheological measurements demonstrated that the nanohybrid hydrogel maintained robust mechanical properties. During frequency sweep analysis from 0.1 to 10 Hz, the storage modulus (G′) of HGM + Multi (∼550 Pa) consistently exceeded the loss modulus (G''), indicating that the hydrogel possessed a predominantly elastic network structure (Fig. 4C). Additional strain dependent measurements (0.01–25%) and thermal cycling (37 °C to 42 °C) experiments confirmed that the hydrogel network remained stable under physiological strain conditions and hyperthermic stimulation, validating its suitability as a stable implant (Fig. S4).

Precise control of MHT allows the tumor temperature to reach 42 to 45 °C, which induces apoptosis in tumor cells while preserving surrounding normal tissues [34]. Moderate hyperthermia can also elevate the expression of heat shock proteins, stimulate antitumor immune responses, and enhance the therapeutic activity of Bev and TMZ [35,36]. We therefore evaluated the magnetothermal properties of the hydrogel and its stimuli responsive drug release behavior. Under an AMF with a current of 6.2 A and a frequency of 245 kHz, the temperature of HGM + Multi rapidly increased from 20 °C to 42 °C within 3 min and then remained stable within the therapeutic hyperthermia range (Fig. 4D and E). The heating performance remained consistent during five consecutive activation cycles, indicating excellent magnetothermal stability (Fig. 4F). Infrared thermographic imaging further confirmed rapid and spatially localized heating in vitro (Fig. 4G and S5).

In a postoperative glioma mouse model, intracavitary administration of the hydrogel increased the local brain temperature by approximately 5 °C during magnetic stimulation, demonstrating effective in vivo heat generation (Fig. 4H). UV–Vis spectroscopy was used to characterize the absorption spectra of TMZ and Bev and to generate calibration curves for quantitative release analysis (Fig. S6). Release studies further showed that the platform exhibited both AMF-responsive and microenvironment-responsive behavior. Under AMF stimulation, payload liberation was markedly accelerated, indicating that magnetothermal activation not only provided local hyperthermia but also facilitated externally regulated drug release from the hydrogel system. Bev release reached approximately 60% within 3 days and nearly 80% within 3 weeks under AMF stimulation, compared with 34% and 63%, respectively, without stimulation. TMZ release similarly increased to approximately 41% at 2 weeks and 52% at 3 weeks under AMF exposure, versus 28% and 39% in the absence of magnetic activation (Fig. 4I). In addition, TMZ release from MT, MTP, and MTPT showed pronounced pH-responsive behavior, with accelerated release under acidic conditions, likely due to degradation of the PDA interfacial layer. Comparable release profiles between MTPT and MTP further indicated that T7 functionalization did not alter acid-triggered release kinetics (Fig. S7). Collectively, these findings support a stimulus-responsive and staged local delivery profile consistent with the intended design of the HGM + Multi(+) platform.

Hydrogel degradation also responded to magnetic stimulation. Approximately 60% degradation occurred within eight days under magnetic activation and nearly complete degradation was observed within three weeks. In contrast, only about 40% degradation occurred after eight days without stimulation and complete degradation required approximately five weeks (Fig. 4J). These release and degradation characteristics ensured that Bev was released during the early stage to inhibit angiogenesis, whereas TMZ was mainly liberated after receptor mediated cellular uptake and subsequent exposure to acidic lysosomal conditions.

The antitumor activity was evaluated using a Transwell coculture model with LN229 glioma cells. While free MTPT suppressed proliferation, HGM + Bev and HGM + Fe groups exhibited negligible cytotoxicity. However, the HGM + Bev + Fe(+) + MTPT group elicited the strongest cytotoxic response after 15 min magnetic stimulation (Fig. 4K and L). Live/dead staining further confirmed extensive tumor cell death in this treatment group, and quantitative analysis across multiple microscopic fields showed that the HGM + Bev + Fe(+) + MTPT group had the highest dead-cell percentage among all groups (Fig. 4M–S8 and S9). These findings demonstrate that the platform enabled cooperative antitumor activity through the combination of MHT and targeted chemotherapy in vitro.

### *3.3* *In vivo* efficacy in preventing GBM relapse

To evaluate therapeutic efficacy in a clinically relevant setting, an orthotopic postoperative GBM model was established using luciferase expressing (Luc^+^) LN229 cells (Fig. S10). Following stereotactic resection of tumors on day 10, mice received intracavitary implantation of the nanohybrid hydrogel (HGM + Multi) (Fig. 5A). Histological examination confirmed the presence of residual tumor cells along the resection margins, which closely resembled the clinical situation of incomplete tumor removal (Fig. 5B and C). The hydrogel exhibited favorable in vivo biodegradation and biosafety profiles. Serial optical images showed progressive dissolution of HGM + Multi(+) from day 1 to week 3, with near-complete degradation by week 3 and no visible swelling or residual material (Fig. S11). Histological examination of major organs, including the heart, liver, spleen, lung, and kidney, revealed normal tissue architecture across all treatment groups without detectable inflammation or necrosis, supporting the absence of acute systemic toxicity (Fig. S12). In addition, in a separate intracranial implantation study using normal C57BL/6J mice with surgically created parenchymal cavities, HGM + Multi(+) did not induce significant astrocytic or microglial activation, as assessed by GFAP and Iba-1 staining, and neuronal integrity identified by NeuN staining remained preserved up to 6 weeks after implantation (Fig. S14).

**Fig. 5:**
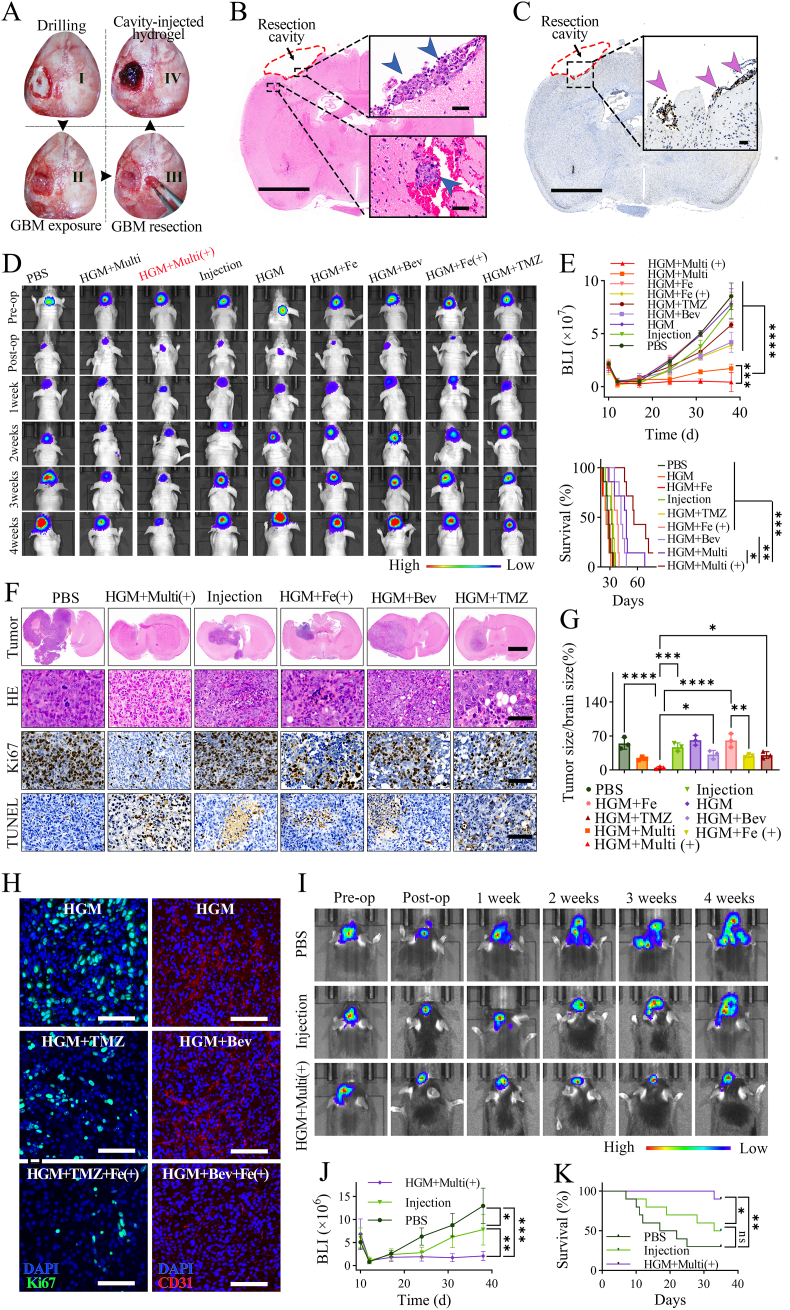
Intracavitary nanohybrid hydrogel suppresses postoperative GBM recurrence and prolongs survival in orthotopic models. (A) Representative photographs of the orthotopic GBM resection procedure, including cranial drilling, tumor exposure, tumor resection, and intracavitary hydrogel injection. (B, C) Representative H&E staining (B) and Ki67 immunohistochemistry (C) of postoperative brain sections showing residual tumor cells adjacent to the resection cavity; insets show enlarged views of the boxed regions. Blue arrows indicate residual tumor cells and purple arrows indicate Ki67-positive cells. (D) Longitudinal bioluminescence imaging (BLI) of tumor progression in the postoperative Luc^+^LN229 orthotopic xenograft model. (E) Top: Quantification of BLI signals over time (mean ± SD, n = 5 biologically independent animals per group). Bottom: Kaplan–Meier survival curves (n = 7 mice per group). Exact pairwise log-rank p-values are provided in Table S1. (F) Representative H&E, Ki67, and TUNEL staining of tumor sections from the indicated groups. (G) Quantification of tumor size (mean ± SD, n = 3 biologically independent animals per group). (H) Immunofluorescence staining of Ki67^+^ glioma cells (green) and CD31^+^ tumor vasculature (red) in the indicated groups, with DAPI counterstaining (blue). (I) Representative BLI images of mice in the PBS, Injection, and HGM + Multi(+) groups in the orthotopic postoperative GL261/C57BL/6J model at the indicated time points. (J) Longitudinal quantification of recurrent tumor burden based on bioluminescence signal intensity in the GL261/C57BL/6J model (mean ± SD, n = 10 mice per group; two-way ANOVA followed by multiple-comparisons testing). (K) Kaplan–Meier survival curves of the indicated groups in the GL261/C57BL/6J model (n = 10 mice per group). Survival differences were analyzed using the log-rank (Mantel–Cox) test; exact pairwise p-values are provided in Table S2. Scale bars, 2 mm in (B, C) main panels; 50 μm in the insets of (B, C); 2 mm in the top row of (F); 100 μm in the lower rows of (F); and 100 μm in (H).

To further evaluate biosafety beyond histological assessment, additional serum biochemical, inflammatory, and elemental analyses were performed. Serum ALT, AST, UA, and Cr levels showed no significant differences among the PBS, Injection, and HGM + Multi(+) groups, suggesting no detectable hepatic or renal toxicity under the tested conditions (Fig. S13A–D). Likewise, serum IL-6 and TNF-α levels remained comparable across groups, indicating the absence of an obvious systemic inflammatory response after implantation (Fig. S13E and F). To assess potential retention of Fe-containing material after substantial hydrogel degradation, ICP-MS was performed at 6 weeks post-operation. No significant difference in Fe content was detected between the Injection and HGM + Multi(+) groups in the brain, liver, or heart (Fig. S13G–I), supporting the lack of detectable abnormal Fe accumulation at this stage. Together, these findings further support the short-to mid-term biosafety of the HGM + Multi(+) platform.

Bioluminescence imaging (BLI) revealed that AMF-activated HGM + Multi(+) produced the strongest suppression of tumor recurrence (P < 0.001 vs. controls). Tumor signals remained markedly lower in this group throughout the observation period. The nonactivated hydrogel also delayed tumor regrowth relative to PBS and nonmagnetic controls, although the effect was less pronounced. MHT clearly amplified the therapeutic efficacy (Fig. 5D). Quantitative analysis of tumor burden confirmed these observations. Kaplan–Meier survival analysis revealed that treatment with magnetically activated HGM + Multi yielded the longest median survival among all experimental groups. Pairwise comparisons were performed using the log-rank test, with exact p-values summarized in Fig. 5E and Table S1. Importantly, this treatment significantly outperformed conventional systemic chemotherapy with intravenous TMZ and Bev. Residual tumor volume was reduced to approximately 5% of the control level. Histological evaluation of H&E-stained brain sections provided additional evidence of therapeutic efficacy. Ki67 and TUNEL immunohistochemistry (IHC) staining confirmed a sharp reduction in proliferative indices and significantly elevated apoptosis in the treatment group (Fig. 5F and G, S15 and S16). Compared with the progressive body weight loss observed in most groups, the HGM + Multi(+) group showed remained stable with a modest upward tendency during treatment (Fig. S17).

GBM progression strongly depends on angiogenesis. Therefore, combining antiangiogenic therapy with chemotherapy and localized MHT represents a rational strategy for controlling tumor recurrence. Under AMF exposure, the implanted hydrogel rapidly reached approximately 42 °C within 3 min (Fig. S18). IHC analysis provided mechanistic insight into this combinatorial effect. HGM + Bev-treated xenografts showed a marked reduction in microvessel density assessed by CD31 staining. Microvessel density dropped to nearly two-thirds of the control level after four weeks of Bev release (Fig. 5H). Additional MHT greatly strengthened this antiangiogenic effect. It reduced microvessel density to roughly one-fourth of control values. Ki67 staining indicated significant inhibition of tumor cell proliferation. Sustained TMZ release decreased the tumor cell proportion to half of control levels. Combined TMZ and MHT treatment further lowered it to one-sixth of control levels (Fig. S19 and S20). Collectively, these results demonstrate that HGM + Multi(+) enables temporally coordinated multimodal therapy that integrates antiangiogenic activity, sustained targeted chemotherapy, and magnetothermal cytotoxicity. This integrated strategy effectively suppresses postoperative GBM recurrence and significantly prolongs survival. To further determine whether the treatment-associated microenvironmental changes observed in the single-cell analysis were accompanied by therapeutic benefit in an immune-competent setting, we next evaluated HGM + Multi(+) in an orthotopic postoperative GL261/C57BL/6J model. Longitudinal bioluminescence imaging showed that recurrent tumor burden increased progressively in the PBS and Injection groups, whereas the HGM + Multi(+) group exhibited a markedly attenuated signal increase over time (Fig. 5I and J). Histological examination at postoperative day 28 further demonstrated a significant reduction in residual tumor area after HGM + Multi(+) treatment (Fig. S21A and B). Consistent with these findings, Kaplan–Meier analysis showed that HGM + Multi(+) significantly prolonged survival relative to control groups (Fig. 5K). Together, these data provide direct efficacy support in the same immunocompetent model used for the scRNA-seq analysis and strengthen the interpretation that the antitumor benefit of HGM + Multi(+) is associated with remodeling of the postoperative tumor microenvironment.

### 3.4 Single-cell transcriptomic landscape and myeloid reprogramming toward pro-inflammatory phenotypes

To assess the impact of multimodal hydrogel therapy on the immune microenvironment, we utilized the immunocompetent GL261/C57BL/6 orthotopic model. Tumor specimens were collected on day 28 from mice treated with HGM + Multi(+) and from injection controls with three biological replicates per group. Single-cell suspensions were processed using 10X Genomics scRNA-seq (Fig. 6A). A total of 67,093 cells were initially captured across all samples. After rigorous quality control filtering, 47,781 high quality cells were retained for downstream analysis (Fig. S22). Unsupervised clustering identified 26 transcriptionally distinct cell clusters, annotated based on canonical markers and copy number variation (CNV) inference including malignant astrocyte-like cells, cancer stem cells (CSCs), endothelial cells, myeloid cells, oligodendrocytes, B-lineage cells, NK cells, pericytes, neutrophils, and T cells. UMAP visualization confirmed the robustness of these annotations and clearly resolved intratumoral heterogeneity (Fig. 6B–G). Quantitative comparison revealed that the TME was dominated by myeloid cells, T cells, and malignant populations. HGM + Multi(+)-treated samples showed a shift in immune composition characterized by increased myeloid representation and reduced T-cell abundance (Fig. 6H), which was also reflected in the corresponding cell counts from the scRNA-seq dataset (Fig. S23 and Table S3).

**Fig. 6:**
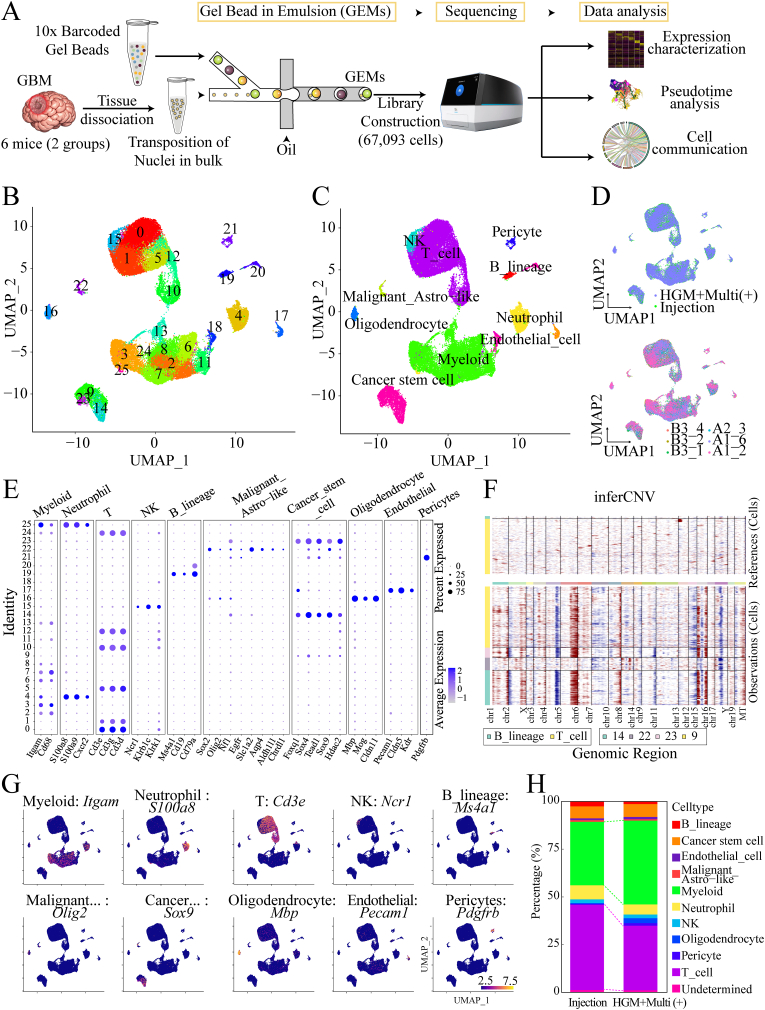
Single-cell transcriptomic landscape of postoperative GBM and treatment-associated cellular composition changes. (A) Schematic overview of the experimental workflow for scRNA-seq analysis of fresh GBM samples, including tissue dissociation, gel bead-in-emulsion (GEM) generation, library construction, sequencing, and downstream bioinformatic analyses. (B) UMAP embedding of 26 transcriptionally distinct cell clusters identified from the integrated GBM scRNA-seq dataset. Each point represents a single cell and colors indicate cluster identity. (C) UMAP visualization of the major annotated cell populations in the GBM microenvironment. (D) UMAP projections colored by treatment group and sample origin, showing the distribution of cells across conditions and samples. (E) Dot plot of canonical marker genes used for cell-type annotation. Dot size represents the percentage of cells expressing each gene, and color intensity indicates the average expression level. (F) inferCNV-based copy-number variation analysis of GBM cells. Red indicates relative copy-number gain and blue indicates relative copy-number loss across genomic regions. Cells are ordered by cluster identity along the y-axis. (G) UMAP feature plots of representative marker genes for selected annotated cell populations. (H) Relative cellular composition of annotated major cell populations in the Injection and HGM + Multi(+) groups within the analyzed scRNA-seq dataset. The percentages shown represent proportional differences among sequenced cells rather than direct measurements of absolute in situ cell abundance. The corresponding annotated immune-cell counts are provided in Fig. S23 and Table S3.

Given the high abundance of myeloid cells in the GBM TME and their central role in tumor progression, immune evasion, angiogenesis, and therapeutic response [37], we investigated the myeloid compartment by re-clustering 18,548 cells into seven transcriptionally distinct subsets, including microglia, monocytes, M1-like macrophages (M1), M2-like macrophages (M2), lipid-associated macrophages (LAM), neutrophils, and proliferating macrophages (Fig. 7A–C). Functional annotation was validated by calculating module scores for M1 (e.g., *Il1b, Tnf, Nos2*), M2 (e.g., *Mrc1, Arg1, Cd163*), and lipid associated macrophages (LAM) signatures (Fig. 7D).

**Fig. 7:**
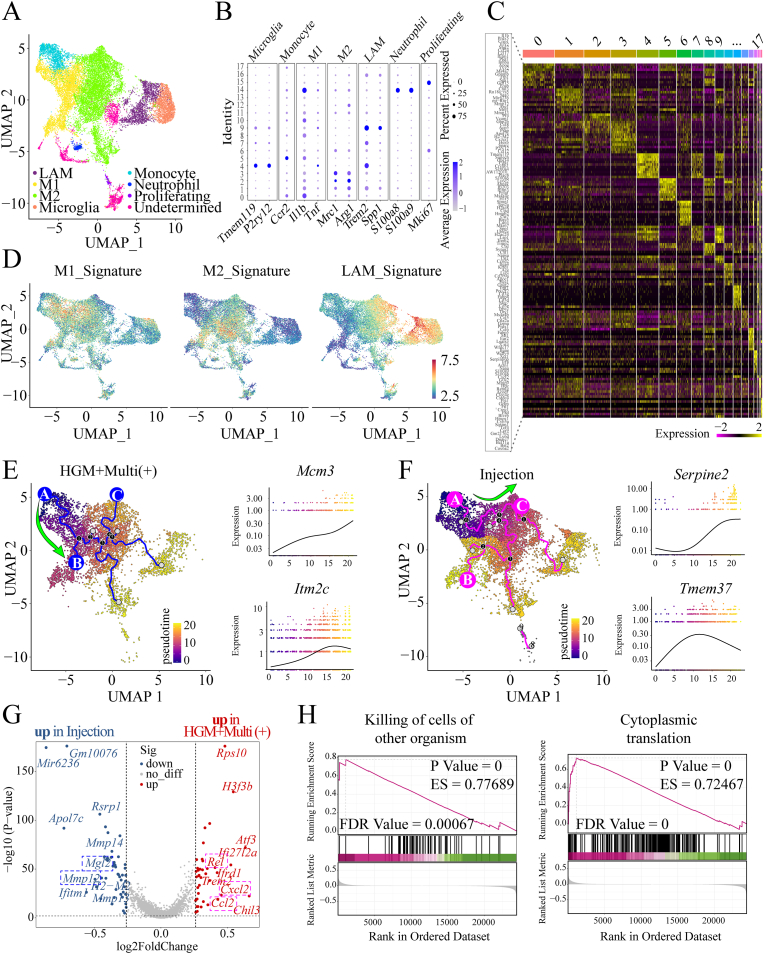
Multidimensional characterization of myeloid subpopulations and differential analysis of treatment groups. (A) UMAP visualization of annotated myeloid subpopulations. (B) Dot plots showing canonical marker genes for distinct myeloid subtypes. (C) Heatmap depicting differential gene expression across myeloid subclusters. (D) UMAP visualization of M1, M2, and LAM signature scores. (E and F) Monocle3 trajectory analysis of myeloid clusters across treatment groups, with green arrows indicating the primary cell-state transition; dynamic expression of the top 2 signature genes (Mcm3/Itm2c and Serpine2/Tmem37, respectively) along pseudotime is shown using a color gradient (dark, early pseudotime; light, late pseudotime). (G) Volcano plot of differentially expressed genes (DEGs) in myeloid cells comparing HGM + Multi(+) versus injection groups. (H) GSEA highlighting pathways significantly enriched in myeloid cells from HGM + Multi(+) versus injection groups.

To elucidate dynamic transitions under therapeutic pressure, pseudotime trajectory analysis via Monocle3 revealed divergent differentiation programs. In HGM + Multi(+)-treated tumors, monocytes (Point A) preferentially progressed toward an M1-like state (Point B). This transition was characterized by sustained high expression of *Mcm3*, which is associated with cell proliferation, and a transient increase in *Itm2c*, which is related to cell adhesion. These transcriptional features are consistent with the emergence of a proliferative and migratory pro-inflammatory phenotype (Fig. 7E). By contrast, monocytes from the injection group predominantly differentiated toward an M2-like state (Point C), characterized by upregulation of *Serpine2* and *Tmem37*, which are associated with tissue repair and anti-inflammatory functions. These transcriptional features are consistent with a more immunoregulatory or pro-reparative macrophage state (Fig. 7F).

These pronounced transcriptomic shifts were corroborated by differentially expressed genes (DEGs) analysis. Myeloid cells from the HGM + Multi(+) group showed significant upregulation of pro-inflammatory genes, including the chemokines *Ccl2* and *Cxcl2*, as well as the *NF-κB* regulator *Rel*. In contrast, macrophages from the injection group showed higher expression of M2-associated genes such as *Mgl2* and *Mmp14* (Fig. 7G). Functional enrichment analysis of the upregulated genes in the treatment group revealed significant enrichment in pathways related to inflammatory response, cytokine activity, and TNF signaling, based on Gene Ontology (GO) and KEGG analyses (Fig. S24). Gene set enrichment analysis (GSEA) further identified activation of pathways related to cytotoxic functions and protein synthesis, including killing of cells of other organisms and cytoplasmic translation (Fig. 7H and S25).

Taken together, these results indicate that HGM + Multi(+) treatment is associated with a shift of myeloid cell states from predominantly immunoregulatory programs toward more pro-inflammatory and biosynthetically active phenotypes. This shift may contribute to a tumor microenvironment that is more permissive to antitumor immune activity while reducing support for tumor persistence. Consistent with this interpretation, the single-cell transcriptomic analysis further revealed coordinated remodeling of the GBM microenvironment, including inflammatory rewiring of myeloid populations, altered T-cell state trajectories, and changes in tumor–stromal communication.

### 3.5 T-cell reinvigoration and reprogramming of intercellular communication

Although T cells showed a relative reduction in abundance, their functional state remains a central determinant of antitumor immunity. We isolated 18,396 T cells and identified six major subtypes including CD4^+^, CD8^+^ CTL, gamma-delta T, Naive/CM T, proliferating T, and Tregs (Fig. S26A–C). UMAP visualization and individual animal analysis confirmed substantial heterogeneity within the T cell compartment (Figs. S26D and S27A, B). Trajectory analysis revealed a pronounced reprogramming of T cell differentiation dynamics. In HGM + Multi(+)-treated tumors, Naive/CM T cells followed a differentiation trajectory distinct from the Treg-skewed path observed in the control group, which was associated with *Hspe1* expression. Instead, these cells maintained high expression of *Mcm3* and progressively upregulated *Ncl*, a regulator of ribosome biogenesis. This pattern is consistent with a transition toward a more activated biosynthetic state (Fig. S26E and F).

Pathway enrichment analysis further supported this functional transition (Fig. S26G and H). T cells in the hydrogel group were enriched in signaling cascades central to activation, including MAPK, NK cell-mediated cytotoxicity, and chemokine signaling. Concomitant enrichment of inflammatory pathways such as IL-17 signaling and ribosome biogenesis reflected a robust biosynthetic state consistent with global activation, as confirmed by GSEA (Fig. S26J and S27C). Taken together, these data suggest that hydrogel treatment alters T-cell state trajectories away from a more suppressive program and toward transcriptional programs consistent with activation, inflammatory signaling, and increased cytotoxic potential. To investigate this effect, we performed ligand receptor interaction analysis using CellChatDB across major cellular populations, including CSCs, T cells, macrophages, oligodendrocytes, and pericytes (Fig. 8A). In injection-treated tumors, CSCs primarily interacted with immune and endothelial populations, which is consistent with mechanisms that support immune evasion. By contrast, HGM + Multi(+) therapy redirected CSC communication toward pericytes and oligodendrocytes (Fig. 8B and S28A, B). This shift may reflect adaptive remodeling of CSC-associated stromal interactions under therapeutic pressure, potentially involving support pathways such as VEGF and PDGF signaling.

**Fig. 8:**
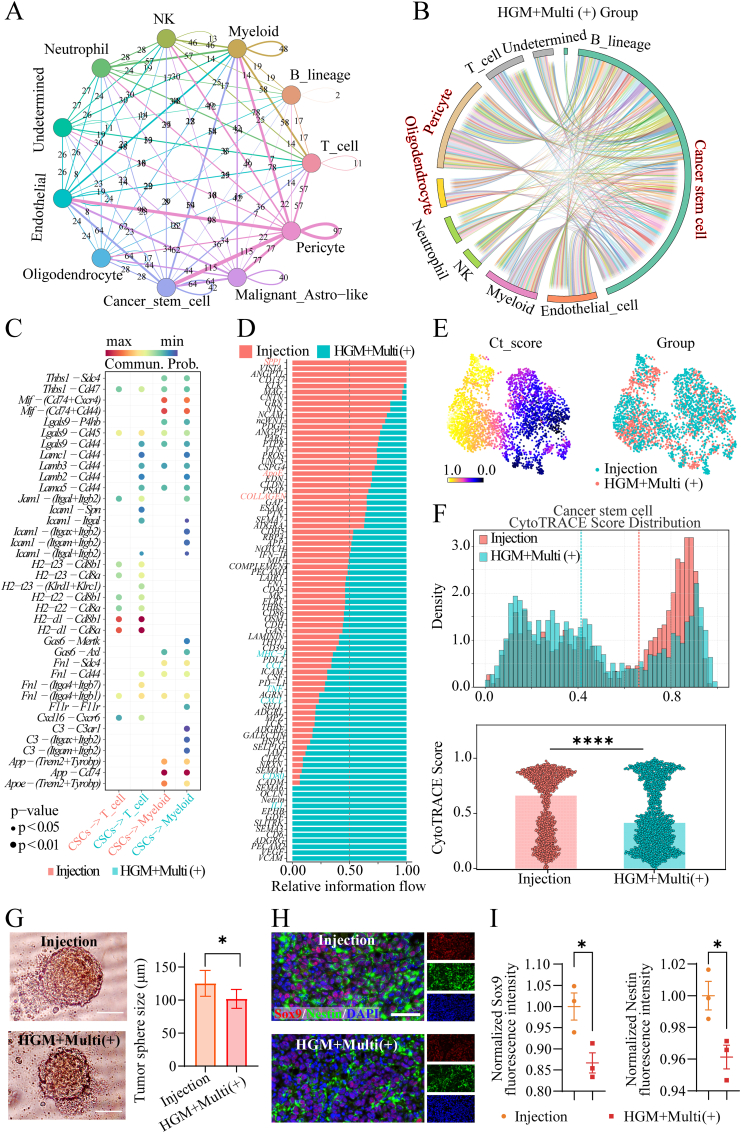
HGM + Multi(+) reprograms tumor intercellular communication and suppresses glioma stemness. (A) Network diagram of significant ligand–receptor interactions among major cell populations. Circle size indicates relative cell abundance and edge width indicates communication strength. (B) CellPhoneDB circular plot for the HGM + Multi(+) group highlighting CSC-centered intercellular communication. (C) Representative ligand–receptor interactions between CSCs and selected cell populations. Dot size is inversely related to the p-value, and color indicates the average expression level. (D) Relative information flow of intercellular communication pathways in the Injection and HGM + Multi(+) groups. (E) UMAP projections of CSCs colored by CytoTRACE score (left) and treatment group (right). (F) Distribution and violin plot of CytoTRACE scores in CSCs from the Injection and HGM + Multi(+) groups. (G) Ex vivo tumorsphere formation by primary cells isolated from recurrent orthotopic GL261 tumors, with representative bright-field images and quantification of sphere diameter. (H) Representative immunofluorescence staining of recurrent GL261 tumor sections for Sox9 (red), Nestin (green), and DAPI (blue). (I) Quantification of normalized Sox9 fluorescence intensity (left) and Nestin fluorescence intensity (right). For each tumor, 3–5 randomly selected fields were analyzed and averaged to generate one value per mouse (n = 3 mice per group). Data are mean ± SD. Statistical significance was determined by unpaired two-tailed Student's t-test. Scale bars, 50 μm in (G, H).

The therapy also reprogrammed CSC-immune interactions (Fig. 8C). Both groups showed MHC-I-mediated antigen presentation, while the signal strength was markedly amplified in HGM + Multi(+), potentially favoring improved T-cell recognition. CSCs additionally upregulated Icam1 and Jam1, which are typically induced by inflammatory cytokines and facilitate stable interactions with myeloid cells. At the network level, macrophage–T-cell interactions were strongly reinforced through ligand–receptor pairs such as Lgals9–Cd44 and Cxcl16–Cxcr6 (Fig. S28C), consistent with enhanced effector-cell recruitment and immune communication. Global information flow analysis revealed that HGM + Multi(+) tumors were dominated by pro-inflammatory pathways including MHC-I, CXCL, and TNF signaling, whereas injection-treated tumors favored pro-tumorigenic fibrosis signals including SPP1 and COLLAGEN (Fig. 8D).

To further evaluate the effect of treatment on tumor cell plasticity, CytoTRACE analysis was performed to estimate differentiation potential based on transcriptional diversity. CSC from the treatment group exhibited a significantly lower stemness score compared with those from the control group, with median values of 0.414 and 0.662 respectively (Fig. 8E and F). These results indicate that hydrogel mediated therapy not only disrupts immunosuppressive signaling networks but also promotes differentiation of CSCs. These findings suggest that the treatment may reduce tumor cell plasticity while disrupting immunosuppressive signaling networks, thereby limiting residual disease progression and postoperative recurrence. Rather than proving a strictly immune-dependent mechanism, these data indicate that the therapy elicits substantial microenvironmental remodeling in immunocompetent tumors that may complement its direct antitumor effects.

To functionally validate the CytoTRACE-predicted reduction in CSC associated stemness, primary tumor sphere formation assays were performed using GBM cells isolated from orthotopic tumors following different treatments. Compared with the PBS and Injection groups, cells derived from HGM + Multi(+) tumors formed significantly fewer and smaller tumor spheres, with markedly reduced sphere formation efficiency (Fig. 8G), indicating impaired self-renewal capacity and stem-like potential after treatment. Consistently, immunofluorescence staining of stemness-associated markers including Sox9 and Nestin, showed reduced expression in tumor tissues from the HGM + Multi(+) group (Fig. 8H and I), further supporting attenuation of CSC-associated features.

## 4 Discussion and conclusion

Glioblastoma recurrence after surgery remains a major clinical challenge because infiltrative residual cells are difficult to eradicate, and the postoperative cavity rapidly evolves into a microenvironment that supports regrowth, vascular remodeling, and therapeutic resistance. In this study, we developed a bioinspired magnetothermal-responsive nanohybrid hydrogel, HGM + Multi(+), as a local multimodal therapeutic platform for postoperative GBM intervention. By integrating sustained local retention, anti-angiogenic modulation, targeted chemotherapy, and AMF-triggered magnetothermal therapy within a single intracavitary depot, this system was designed to address both the residual tumor cells that escape resection and the recurrence-prone niche that supports their expansion.

A notable feature of this platform is its hierarchical and temporally coordinated therapeutic design. Bev was preferentially released at an early stage to inhibit angiogenesis in the postoperative microenvironment, whereas TMZ was mainly liberated after receptor-mediated cellular uptake and subsequent exposure to acidic lysosomal conditions. In parallel, incorporation of superparamagnetic Fe_3_O_4_ nanoparticles enabled externally triggered heat generation under AMF stimulation, thereby providing both direct magnetothermal cytotoxicity and accelerated therapeutic release. PDA served as a versatile interfacial coating for drug loading and nanosystem assembly, while T7 peptide functionalization supported glioma-directed uptake through transferrin receptor-associated targeting. Together, these features allowed HGM + Multi(+) to function not simply as a passive carrier, but as an integrated local therapeutic system with programmable and complementary modes of action.

Our in vitro results support this design rationale. Although free MTPT alone suppressed LN229 cell proliferation, formulations lacking either the complete therapeutic payload or AMF activation showed limited cytotoxicity, whereas HGM + Bev + Fe(+) + MTPT elicited the strongest tumor cell killing after magnetic stimulation. These findings indicate that therapeutic efficacy depends on the coordinated contribution of magnetothermal treatment and targeted chemotherapy rather than on any single component alone. More broadly, they suggest that spatially confined heat generation can potentiate local drug action in residual glioma cells, which is particularly relevant in the postoperative setting where infiltrative cells may remain dispersed along the resection margin.

The in vivo data further demonstrate the translational relevance of this strategy. In the orthotopic postoperative LN229 model, intracavitary implantation of HGM + Multi(+) after tumor resection markedly suppressed tumor recurrence and significantly prolonged survival. The hydrogel also exhibited favorable short-to mid-term biodegradation and biosafety profiles, with progressive dissolution over time, no visible swelling or residual bulk material by week 3, and no obvious pathological abnormalities in major organs under the tested conditions. These findings support the feasibility of using this HAMA/GelMA-based local matrix as a postoperative depot capable of maintaining therapeutic exposure within the resection cavity while avoiding the limitations imposed by the blood–brain barrier.

Importantly, the therapeutic effect of HGM + Multi(+) appears to extend beyond direct cytotoxicity alone. Our single-cell transcriptomic analysis in the immunocompetent postoperative GL261/C57BL/6J model showed that treatment was associated with marked reprogramming of myeloid populations, altered T-cell differentiation trajectories, and broad rewiring of ligand–receptor communication. These changes support the view that the hydrogel system not only damages tumor cells locally but also reshape the postoperative tumor ecosystem. The newly added efficacy data in the same GL261/C57BL/6J model further strengthen this interpretation, as HGM + Multi(+) was associated with reduced recurrent tumor burden and prolonged survival under immune-intact conditions. Taken together, these findings suggest that the overall therapeutic benefit of this platform reflects the integrated outcome of direct local antitumor effects together with immune-context-dependent remodeling of the microenvironment.

At the same time, the current data also help refine the interpretation of model-dependent findings. In the LN229/BALB/c nude model, the observed efficacy primarily reflects immune-independent functions of the platform, including local magnetothermal cytotoxicity, TMZ-mediated chemotherapy, sustained postoperative retention, and Bev-associated anti-angiogenic effects. Because adaptive immunity is impaired in this host background, these experiments were not intended to establish T-cell-dependent mechanisms. By contrast, the syngeneic GL261/C57BL/6J model enabled assessment of treatment-associated host responses in the presence of an intact immune system. The shorter absolute survival window observed in GL261/C57BL/6J mice compared with LN229-bearing nude mice should therefore not be interpreted as evidence against an immune contribution or as a contradiction of the mechanistic findings. Rather, the two models differ fundamentally in tumor origin, host background, growth kinetics, and baseline immunological context, all of which can influence disease course and therapeutic responsiveness. Within this framework, the cross-model results are better viewed as complementary: the LN229 model supports the robust local efficacy of the platform in a clinically relevant postoperative setting, whereas the GL261 model provides evidence that this efficacy can coexist with, and potentially be reinforced by, treatment-associated immune remodeling.

Another important aspect of the present study is the potential impact of treatment on stem-like tumor cell states. Our results from tumor sphere-related assays suggest that HGM + Multi(+) may suppress biological features associated with glioma stemness, which is noteworthy because cancer stem-like cells are widely implicated in GBM recurrence, treatment resistance, and tumor re-establishment after surgery. In the postoperative context, this point is particularly relevant: even when bulk tumor mass is largely removed, a small population of stem-like residual cells may remain at infiltrative margins and serve as a reservoir for relapse. Thus, the observed reduction in sphere-forming capacity, together with the in vivo recurrence suppression data, supports the possibility that this multimodal local therapy affects not only proliferating bulk tumor cells but also cellular states linked to long-term regenerative potential. That said, while these findings are biologically suggestive, they should not yet be interpreted as definitive proof of durable CSC elimination or irreversible lineage restriction. Future studies incorporating lineage tracing, serial sphere passaging, and limiting-dilution transplantation assays will be important to determine whether HGM + Multi(+) induces stable long-term changes in CSC fate and tumor-propagating capacity.

From a biomaterials perspective, this work also highlights the value of combining structural retention with stimulus responsiveness in postoperative brain tumor therapy. Conventional local drug delivery systems can improve regional exposure, but their therapeutic scope is often constrained by passive release, limited targeting, and insufficient activity against infiltrative residual disease. Here, the nanohybrid hydrogel was designed to remain within the resection cavity, provide sustained local exposure, and respond to external magnetic stimulation, thereby creating opportunities for repeated or controllable intervention after implantation. This design is especially relevant to GBM, where residual cells are spatially heterogeneous and difficult to eradicate using conventional local delivery alone. By coupling local cytoreduction with modulation of angiogenic and immune-associated features of the postoperative niche, HGM + Multi(+) expands the conceptual role of intracavitary biomaterials from drug reservoirs to active regulators of the recurrence microenvironment.

Several limitations should nevertheless be acknowledged. First, although we now provide both efficacy and mechanistic observations in the same immunocompetent GL261/C57BL/6J model, the present study does not constitute a fully unified causal analysis linking specific immune cell populations to therapeutic benefit. Therefore, our data support an association between treatment efficacy and immune remodeling, but do not yet establish the precise contribution of individual immune subsets or signaling axes. Second, while the single-cell transcriptomic analysis provides valuable mechanistic insight, the number of biological replicates for scRNA-seq remains limited and the findings should be interpreted in conjunction with histological and in vivo efficacy data. Third, a more rigorous dissection of combinatorial benefit would require dedicated comparison of matched single-component, dual-component, and full-combination formulations under identical experimental conditions, which was beyond the scope of the current study. Accordingly, the observed antitumor effects are described as resulting from an integrated multimodal mechanism rather than from formally established component synergy. Fourth, although the hydrogel showed favorable short-to mid-term intracranial biodegradation and biosafety in the current experiments, the long-term fate of Fe_3_O_4_-containing residues and the possibility of nanoparticle persistence in the brain warrant further investigation. Finally, future integration of spatial transcriptomics and multiplex tissue imaging would help define where treatment-induced cellular interactions occur within the postoperative tumor bed, particularly at infiltrative margins and in perivascular regions.

In conclusion, HGM + Multi(+) functions as a multimodal local therapeutic platform for postoperative GBM intervention. Our findings support a hierarchical mode of action in which direct magnetothermal, chemotherapeutic, and anti-angiogenic effects are accompanied by substantial remodeling of the tumor microenvironment under immune-competent conditions. At the same time, the observed suppression of tumor sphere associated phenotypes suggests that this strategy may also interfere with stem-like residual cell states that contribute to relapse. Although further work is needed to establish long term causal mechanisms and CSC fate outcomes, the present study provides evidence that intelligent intracavitary biomaterials can be engineered not only to kill residual tumor cells locally, but also to reshape the biological context in which recurrence emerges. This integrated framework may offer a promising direction for the development of next-generation postoperative therapies for GBM.

## Data availability

Data are available when requested to the authors.

## Ethics approval and consent to participate

All procedures using female BALB/c nude mice (4–6 weeks old, RRID: IMSR_CRL:022) and C57BL/6J mice (4–6 weeks old, RRID: IMSR_JAX:000664) obtained from the Zhejiang Provincial Center for Laboratory Animals were approved by the Ethical Committee of Wenzhou Institute, University of Chinese Academy of Sciences (WIUCAS24092301), and the study was conducted in compliance with the Public Health Service (PHS) Policy on Humane Care and Use of Laboratory Animals, ARRIVE Guidelines 2.0 (Percie du Sert et al., PLoS Biol, 2020), and the Laboratory Animal Care and Use Guidelines.

## CRediT authorship contribution statement

**Nan Wu:** Data curation, Visualization, Writing – original draft. **Xi Luan:** Methodology. **Bihe Yuan:** Data curation. **Mingyuan Xia:** Formal analysis. **Jianliang Shen:** Project administration. **Jing Lin:** Data curation. **Lixia Xu:** Resources. **Hai Lin:** Visualization, Writing – review & editing. **Lexiang Zhang:** Project administration, Writing – review & editing. **Fangfu Ye:** Project administration, Writing – review & editing. **Hua Yan:** Resources, Writing – review & editing.

## Declaration of competing interest

Jianliang Shen is an editorial board member for Bioactive Materials and was not involved in the editorial review or the decision to publish this article. All authors declare that there are no competing interests.
