# Supplementary material for: Single-cell transcriptome-guided biomimetic magnetothermal hydrogel microspheres for multimodal eradication of residual glioblastoma
Source: Bioact Mater. 2026 Jul 28;66:1115–32. doi: 10.1016/j.bioactmat.2026.07.043 (PMC13450493; doi:10.1016/j.bioactmat.2026.07.043)
Supplement: Supplementary file 1 [file mmc1.docx]

Supporting Information

**Single-cell Transcriptome-guided Biomimetic** **Magnetothermal Hydrogel Microspheres for Multimodal Eradication of Residual Glioblastoma**

**Fig. S1**. (A-B) Representative TEM, EDS, and SEM images, along with particle size distributions of modified MSNs. Scale bars, 100 nm in (A and B).

**Fig. S2**. Characterization of Fe₃O₄: (A) Different states of Fe₃O₄ NPs: powder, suspension, and magnetic responsiveness. (B) TEM image and magnified view (inset). (C) Particle size distribution histogram. (D) Lattice fringes of Fe₃O₄. (E) EDS spectrum confirming its elemental composition (Fe, O). Scale bars: 100 nm in (B), 50 nm in the inset of (B), 1 nm in (D), 50 nm in (E).

**Fig. S3.** Effect of Fe₃O₄ content on the magnetothermal behavior and extrusion performance of the hydrogel. (A) Temperature elevation profiles of hydrogels containing different Fe₃O₄ concentrations (30, 70, 100, and 150 mM) under AMF exposure (245 kHz, 6.2 A). Increasing Fe₃O₄ content progressively enhanced the heating efficiency of the hydrogel. (B) Representative photographs showing the extrusion behavior of hydrogels with different Fe₃O₄ concentrations under identical conditions. Increasing Fe₃O₄ content affected filament continuity and extrusion morphology.

**Fig. S4.** Rheological Tests of HGM+Multi and HAMA: Amplitude Sweeps (0.01–25% Shear Strain, 1 Hz) and Temperature-Time Sweeps (37→42°C, 300 s Duration).

**Fig. S5.** Infrared thermal images of the HGM+ Fe_3_O_4_ and HGM+Bev+TMZ under AMF (6.2 A, 245 kHz; coil: 100 mm inner diameter, 6 turns).

**Fig. S6**. UV-Vis spectra of TMZ (A) and Bev (B) and their corresponding standard curves.

**Fig. S7**. In vitro TMZ release kinetics from (A) MT, (B) MTP, and (C) MTPT nanoparticles under physiological and acidic conditions.

**Fig. S8.** Live/dead staining of LN229 cells treated with indicated components ± AMF (24 h). All data are mean ± SD (n = 3). Scale bars: 100 µm.

**Fig. S9.** Quantitative analysis of live/dead staining in different treatment groups. (A) Quantification of the proportions of live and dead cells in each group based on fluorescence images analyzed using ImageJ. (B) Statistical analysis of dead cell percentages in the indicated groups. Live/dead staining images were acquired under identical imaging settings, and multiple representative microscopic fields were analyzed for each group. Data are presented as mean ± SD. Statistical significance was determined by one-way ANOVA with Tukey’s multiple-comparisons test.

**Fig. S10.** Study design and treatment timeline.

**Fig. S11.** Optical images depicting the dissolution of the nanohybrid hydrogel HGM+Multi applied to the mouse brain.

**Fig. S12.** H&E staining analyses of major organs after different treatments. Scale bars: 100 μm.

**Fig. S13.** Systemic biosafety evaluation and tissue iron analysis after treatment. (A–D) Serum biochemical analysis of alanine aminotransferase (ALT, A), aspartate aminotransferase (AST, B), creatinine (Cr, C), and uric acid (UA, D) in the PBS, Injection, and HGM+Multi(+) groups.

(E–F) Serum levels of inflammatory cytokines IL-6 (E) and TNF-α (F) measured by ELISA.

(G–I) ICP-MS quantification of Fe concentration in the brain (G), liver (H), and heart (I) at 6 weeks after implantation in the PBS and HGM+Multi(+) groups. Data are presented as mean ± SD. Statistical analysis was performed using one-way ANOVA with Tukey’s multiple-comparisons test for A–F, and unpaired two-tailed Student’s t-test for G–I.

**Table S1.** Exact log-rank p-values for pairwise comparisons of survival curves shown in Fig. 5F.

| Pairwise comparison | Log-rank p-value | Median survival, first group (days) | Median survival, second group (days) |
| --- | --- | --- | --- |
| PBS vs HGM+Multi(+) | 0.0001 | 28.0 | 56.0 |
| HGM vs HGM+Multi(+) | 0.0001 | 26.0 | 56.0 |
| HGM+Fe vs HGM+Multi(+) | 0.0001 | 29.0 | 56.0 |
| Injection vs HGM+Multi(+) | 0.0002 | 32.0 | 56.0 |
| HGM+Fe(+) vs HGM+Multi(+) | 0.0002 | 38.0 | 56.0 |
| HGM+TMZ vs HGM+Multi(+) | 0.0002 | 33.0 | 56.0 |
| HGM+Bev vs HGM+Multi(+) | 0.0012 | 42.0 | 56.0 |
| HGM+Multi vs HGM+Multi(+) | 0.0361 | 48.0 | 56.0 |

**Note:** Pairwise comparisons of Kaplan–Meier survival curves were performed using the log-rank (Mantel–Cox) test. Exact p-values are shown for all comparisons. Median survival is reported in days.

**Fig. S14.** Local brain biocompatibility of HGM+Multi(+) after implantation into a normal brain parenchymal cavity. (A) Representative H&E-stained brain sections at 6 weeks after implantation of PBS or HGM+Multi(+) into a small parenchymal cavity in normal C57BL/6J mice. No obvious disruption of the surrounding tissue architecture was observed in the HGM+Multi(+) group compared with PBS controls. Scale bars, 200 μm (upper row) and 1 mm (lower row).

(B) Representative immunofluorescence images of GFAP, Iba-1, and NeuN in the peri-cavity brain region at 6 weeks post-implantation, together with quantitative analysis of GFAP-positive area fraction, Iba-1-positive area fraction, and NeuN-positive neuronal density. No significant differences were detected between PBS and HGM+Multi(+) groups. Each dot represents one ROI from 3–5 mice per group. Data are presented as mean ± SD. Statistical analysis was performed using an unpaired two-tailed Student’s t-test. Scale bars, 100 μm.

**Fig. S15.** Representative H&E-stained images from each group showing tumor growth and histopathological analysis of tumor sections showing H&E staining results, proliferation index (Ki67), and apoptosis (TUNEL assay). Scale bars: 2 mm in the top row, 100 μm in rows 2, 3, and 4.

**Fig. S16.** Quantitative analysis of ki67 and TUNEL. (n = 3 biologically independent animals per group). (one-way ANOVA with Tukey's post-hoc test).

**Fig. S17.** The changes in body weight of the mice bearing resection glioma with each treatment. The data are shown as the mean ± SD. (n = 5 biologically independent experiments per group). The data are representative results of three experiments.

**Fig. S18**. Infrared thermography of the implanted HGM+Multi(+) hydrogel during AMF exposure (3 min), with insets depicting pre- and post-stimulation temperature profiles. Scale bars: 5 mm.

**Fig. S19.** Representative fluorescent micrographs and quantitative analysis of relative vascular density in tumors via endothelial marker CD31 (red) staining, in different treatment groups. All data are presented as means ± SD. For comparisons among three or more groups, one-way analysis of variance (ANOVA) followed by post hoc Bonferroni test was used. DAPI ,4′,6-diamidino-2-phenylindole. Scale bars: 100 µm.

**Fig. S20.** Representative fluorescent micrographs and quantitative analysis of proliferative index in tumors via proliferation marker Ki67 (green) staining, in different treatment groups. All data are presented as means ± SD. For comparisons among three or more groups, one-way analysis of variance (ANOVA) followed by post hoc Bonferroni test was used. DAPI ,4′,6-diamidino-2-phenylindole. Scale bars: 100 µm.

**Table S2.** Exact log-rank p-values for pairwise comparisons of survival curves shown in Fig. 5K.

| Pairwise comparison | Log-rank p-value | Median survival, first group (days) | Median survival, second group (days) |
| --- | --- | --- | --- |
| PBS vs HGM+Multi(+) | 0.0035 | 19.5 | Undefined |
| Injection vs HGM+Multi(+) | 0.0427 | 34.0 | Undefined |
| PBS vs Injection | 0.2092 | 19.5 | 34.0 |

**Note:** Pairwise comparisons of Kaplan–Meier survival curves were performed using the log-rank (Mantel–Cox) test. Exact p-values are shown for all comparisons. Median survival is reported in days.

**Fig. S21.** HGM+Multi(+) is associated with reduced recurrent tumor burden by histological analysis. (A) Representative H&E-stained coronal brain sections from the PBS, Injection, and HGM+Multi(+) groups collected at the study endpoint. Scale bar, 1 mm. (B) Quantification of tumor burden expressed as tumor size/brain size (%). Each dot represents one mouse (n = 5 mice per group). Data are presented as mean ± SD. Statistical analysis was performed using one-way ANOVA followed by Tukey’s multiple comparisons test.

**Fig. S22.** Data distribution of single-cell RNA sequencing (scRNA-seq) samples before and after quality control.

**Table S3.** Numbers of annotated cell populations identified in the scRNA-seq dataset from the Injection and HGM+Multi(+) groups

| Cell type | Injection (cell count) | HGM+Multi(+) (cell count) |
| --- | --- | --- |
| B_lineage | 596 | 365 |
| Cancer stem cell | 1413 | 1691 |
| Endothelial_cell | 228 | 311 |
| Malignant_Astro-like | 191 | 176 |
| **Myeloid** | **7509** | **11039** |
| Neutrophil | 1670 | 1322 |
| NK | 441 | 493 |
| Oligodendrocyte | 102 | 637 |
| Pericyte | 102 | 345 |
| **T_cell** | **10102** | **8517** |
| Undetermined | 270 | 261 |

**Fig. S23.** Bar plot showing the numbers of captured myeloid cells and T cells in the scRNA-seq dataset from the Injection and HGM+Multi(+) groups. This analysis was added to complement the proportional changes shown in Fig. 6H. The HGM+Multi(+) group contained more myeloid cells and fewer T cells than the Injection group within the sequenced dataset.

**Fig. S24**. GO and KEGG pathway enrichment of myeloid cells DEGs (HGM+Multi(+) vs. injection).

**Fig. S25**. GSEA of myeloid cells DEGs between HGM+Multi(+) and injection groups.

**Fig. S26.** T-cell transition from immunosuppressive trajectory to hyperactivation under HGM+Multi(+) therapy. (A) UMAP embedding and unsupervised clustering of T-cells, colored by identified T-cell subtypes. (B) Dot plots of canonical marker genes used to define each T-cell subtype. (C) Heatmap showing DEGs across T-cell clusters. (D) UMAP feature plots showing representative marker genes for selected T-cell clusters. (E-F) Pseudotemporal trajectory of T-cell states inferred by Monocle3. Dynamic expression of the top two signature genes (Mcm3/Ncl and Mcm3/Hspe1, respectively) per T-cell clusters and UMAP projections of these genes across treatment groups. (G) GO functional enrichment analysis and (H) KEGG pathway enrichment analysis of T-cells DEGs between HGM+Multi(+) and injection groups. (J) GSEA highlighting pathways significantly enriched in T cells from HGM+Multi(+) versus injection groups.

**Fig. S27**. (A-B) UMAP visualizations of T cells stratified by samples and treatment origins. (C) GSEA of T cells DEGs between HGM+Multi(+) and injection groups.

**Fig. S28**. (A) A circular plot from CellPhoneDB for the injection group, emphasizing intercellular communication centered on CSCs. Colored chords connect various cell types to ligand-receptor interactions involving CSCs. (B) Ligand-receptor pairs from CellPhoneDB for HGM+Multi(+) group, emphasizing intercellular communication centered on CSCs. (C) Interactions between two cell clusters via ligand - receptor pairs, larger dots correspond to smaller p–values, and the color gradient from dark blue to red reflects increasing average expression levels.
